# Combating multidrug-resistant bacteria and associated virulence factors using *Cichorium intybus* extract: integrated microbiological characterization, phytochemical profiling, cytotoxicity assessment, and mechanistic insights

**DOI:** 10.1038/s41598-026-53690-2

**Published:** 2026-05-26

**Authors:** Mohamed Ibrahim M. Ramadan, Gamal M. El-Sherbiny, Ahmad S. El-Hawary, Mostafa M. Basuoni

**Affiliations:** Botany and Microbiology Department, Faculty of Science, Al-Azhar University, Cairo, 11884 Egypt

**Keywords:** Clinical specimens, MDR bacteria, Antibacterial activity, Antibiofilm activity, Antioxidant activity, Cytotoxicity, Apoptosis induction, *C. intybus*, Phytochemical analysis, GC–MS, HPLC, Drug discovery, Microbiology

## Abstract

The global emergence of multidrug-resistant (MDR) Gram-negative pathogens necessitates novel therapeutic agents targeting bacterial growth and virulence. This study investigated the antibacterial, antibiofilm, antioxidant, cytotoxic, and apoptosis-inducing activities of *Cichorium intybus* leaf extract against MDR clinical isolates, alongside phytochemical profiling. Seventy-five clinical specimens were analyzed, predominantly blood (24%), sputum, pus, and urine (20% each), with stool samples underrepresented (4%; χ^2^(4) = 12.12, *p* = 0.033). A total of 75 bacterial isolates were identified using morphology, biochemical testing, and VITEK^®^2 (96–98% accuracy), including *Klebsiella pneumoniae* (56%), *Escherichia coli* (24%), and *Acinetobacter baumannii* (20%) (χ^2^(2) = 17.52, *p* < 0.001). The isolates exhibited high resistance to β-lactams, moderate resistance to aminoglycosides and tetracyclines, and greater susceptibility to imipenem and amikacin (χ^2^(3) = 92.4, *p* < 0.001). The *C. intybus* extract exhibited notable antibacterial activity, with the largest inhibition zone observed against *K. pneumoniae* (19.95 ± 2.16 mm). The minimum inhibitory concentration (MIC) and minimum bactericidal concentration (MBC) values ranged from 31.25 to 187.5 µg/mL and 62.5 to 375 µg/mL, respectively. Biofilm formation by *A. baumannii*, *E. coli*, and *K. pneumoniae* was significantly inhibited in a dose-dependent manner (0.78–50 µg/mL), with maximal suppression at 50 µg/m. Antioxidant assays demonstrated strong activity, with 92.2 ± 1.5% DPPH and 90.5 ± 2.0% ABTS inhibition at 1000 µg/mL, and IC_50_ values of ~ 110 and ~ 115 µg/mL, respectively, showing strong correlation with ascorbic acid (*r* = 0.997 and 0.995; *p* < 0.05). Cytotoxicity assays revealed selective, dose-dependent activity against PC3 and HepG2 cells compared with normal HFB4 cells (IC_50_ = 24.6, 21.9, and 59.7 µg/mL, respectively), with apoptosis as the primary mechanism and minimal necrosis at lower doses. GC–MS and HPLC analyses identified bioactive compounds including chlorogenic, cichoric, linoleic, hexadecanoic, and octadecanoic acids, supporting synergistic antimicrobial, antioxidant, and anticancer effects. Overall, *C. intybus* demonstrates promising multifunctional bioactivity against MDR pathogens, supporting its potential as a natural therapeutic candidate.

## Introduction

Multidrug resistance constitutes a critical and escalating global health crisis that severely undermines the efficacy of contemporary antimicrobial therapy. MDR arises when microorganisms-predominantly bacteria-acquire resistance to multiple structurally and mechanistically distinct classes of antimicrobial agents, thereby rendering standard therapeutic regimens ineffective and contributing to prolonged morbidity, elevated mortality, and substantial economic burden^1,2^. The World Health Organization has designated antimicrobial resistance (AMR) among the foremost threats to global health, estimating that in 2019 alone, resistant infections were associated with approximately 4.95 million deaths worldwide, including 1.27 million deaths directly attributable to bacterial AMR^3,4^. Clinically significant MDR pathogens-including, *Klebsiella pneumoniae*,* Pseudomonas aeruginosa*,*Acinetobacter baumannii* and methicillin resistant *Staphylococcus aureus*-are implicated in severe hospital- and community-acquired infections such as pneumonia, bacteremia, urinary tract infections, chronic wound infections, and device-associated biofilm-related diseases. The proliferation of these organisms threatens the safety of surgical interventions, cancer chemotherapy, organ transplantation, and critical care medicine, all of which depend on effective antimicrobial prophylaxis and treatment. Projections further suggest that, without coordinated global intervention, AMR could account for up to 10 million deaths annually by 2050, exceeding cancer-related mortality and imposing profound socioeconomic consequences^4^.

In this context, a substantial proportion of nosocomial *Klebsiella* spp., particularly carbapenem-resistant *K. pneumoniae*, are associated with mortality rates approaching 50%, which may exceed 70% in cases involving polymyxin resistance. Carbapenem-resistant *A. baumannii* has likewise emerged as a predominant etiological agent of ventilator-associated pneumonia in Indian intensive care units. As one of the largest global consumers of antibiotics, India faces accelerated selection and dissemination of resistant strains, reinforcing the urgent need for innovative therapeutic strategies as emphasized by the World Health Organization^4,5^. Central to bacterial pathogenicity are quorum sensing (QS) systems and biofilm formation-highly coordinated regulatory networks governing collective behaviors such as toxin production, adhesion, immune evasion, and antibiotic tolerance^6^. Conventional antibiotics predominantly target bacterial viability and often fail to disrupt these virulence-associated mechanisms; in certain contexts, subinhibitory exposure may even promote adaptive resistance and enhanced pathogenicity.

Accordingly, therapeutic strategies that target the attenuation of microbial virulence, rather than direct inhibition of bacterial growth, have increasingly been recognized as a promising next-generation approach to anti-infective intervention^1,7^. In this context, phytochemical-rich medicinal plants constitute a largely underexploited reservoir of structurally diverse bioactive metabolites with broad-spectrum antimicrobial potential^8^. Unlike conventional antibiotics, plant-derived secondary metabolites—including phenolic acids, flavonoids, terpenoids, and alkaloids—exert pleiotropic effects through modulation of key virulence-associated pathways, such as quorum sensing (QS), biofilm development, efflux pump activity, and membrane integrity^9–12^. Notably, phenolic constituents, including chlorogenic and caffeic acid derivatives, have demonstrated pronounced QS inhibitory and antibiofilm activities against *Pseudomonas aeruginosa*^12,13^. Collectively, these multitarget antivirulence mechanisms may mitigate selective pressure for resistance development while enhancing the efficacy of conventional antimicrobial therapies^7–10^.

Among medicinal plants, *Cichorium intybus* L. (chicory), a member of the *Asteraceae* family, has attracted increasing scientific interest due to its rich and chemically diverse phytoconstituent profile^14^. Phytochemical investigations have identified abundant flavonoids, phenolic acids (notably chlorogenic and caffeic acids), coumarins, and sesquiterpene lactones, which collectively underpin its documented antioxidant, antimicrobial, and anti-inflammatory properties^15,16^. Despite growing interest in *C. intybus*, critical gaps remain, including limited focus on virulence mechanisms, insufficient advanced phytochemical characterization, and scarce integration of composition–activity relationships against MDR pathogens. This study addresses these limitations by evaluating the antibacterial and anti-virulence effects of *C. intybus* against MDR clinical isolates, including impacts on quorum sensing and biofilm formation, alongside antioxidant and apoptosis-modulating activities. Comprehensive phytochemical profiling is also performed to elucidate potential mechanistic links, thereby supporting its potential as a multifunctional agent targeting both antimicrobial resistance and virulence.

## Materials and methods

### Chemicals and reagents

All antibacterial agents, antioxidant standards, and reference chemicals were procured from (Sigma-Aldrich and HiMedia Laboratories India). Microbiological culture media were obtained exclusively from (Oxoid, UK). All other reagents and solvents employed throughout the study were of analytical grade and used without further purification.

### Study design and methodological workflow

The study was designed as a two-phase investigation: (1) collection of clinical samples, followed by microbiological identification and comprehensive antibiotic susceptibility profiling of the recovered bacterial isolates; and (2) collection of *C. intybus* leaves, subsequent extraction, and systematic evaluation of their antimicrobial activity, cytotoxic potential, and phytochemical composition.

#### Collection of clinical specimens

Between March 2024 and April 2025, a total of seventy-five clinical specimens were collected from patients admitted to Qasr El Aini Hospitals, Cairo, Egypt. The specimens comprised pus, urine, blood, sputum, stool, and central venous catheter (CVC) samples, all of which were obtained and processed under strict aseptic conditions for subsequent microbiological analysis.

Clinical sample collection was conducted following acquisition of written informed consent from all participants. The study protocol was reviewed and approved by the Institutional Review Board of the National Cancer Institute, Cairo University, Egypt (IRB No.: IRB-00004025; approval No.: CB2410-102-083-192), ensuring full compliance with established ethical standards for research involving human subjects.

#### Isolation and identification bacterial isolates

Clinical specimens were aseptically inoculated onto MacConkey agar and blood agar plates (Oxoid, UK).) and incubated aerobically at 37 °C for 24 h. Following incubation, well-isolated colonies were subcultured on the corresponding media under identical conditions to obtain pure bacterial isolates. Preliminary identification was performed using conventional microbiological approaches, including colony morphology, Gram staining, and standard biochemical assays. Definitive identification was subsequently achieved using the VITEK^®^ 2 automated identification system (bioMérieux, Marcy-l’Étoile, France) in accordance with the manufacturer’s instructions.

#### Antibiotics susceptibility and selection of MDR bacterial isolates

A total of 75 purified bacterial isolates were evaluated for susceptibility to eight antimicrobial agents representing different antibiotic classes: penicillins (penicillin G, 10 IU), cephalosporins (cefuroxime, 30 µg; cefaclor, 30 µg), aminoglycosides (amikacin, 30 µg; tobramycin, 10 µg), carbapenems (imipenem, 10 µg), tetracyclines (doxycycline, 30 µg), and polymyxins (colistin). All antimicrobial disks were obtained from Oxoid (UK). Susceptibility testing for all antibiotics except colistin was performed using the Kirby–Bauer disk diffusion method on Mueller–Hinton agar plates. Bacterial suspensions were adjusted to 0.5 McFarland standard and uniformly inoculated onto the agar surface. Plates were incubated at 37 °C for 18–24 h, after which inhibition zone diameters were measured and interpreted as susceptible (S), intermediate (I), or resistant (R) according to the guidelines of the Clinical and Laboratory Standards Institute.

Because disk diffusion methods are unreliable for polymyxins, colistin susceptibility was determined using the broth microdilution method in cation-adjusted Mueller–Hinton broth following CLSI recommendations. Two-fold serial dilutions of colistin were prepared in sterile 96-well microtiter plates to determine the MIC. Bacterial inocula equivalent to 0.5 McFarland standard were added to each well and plates were incubated at 37 °C for 18–20 h. The MIC was defined as the lowest concentration of colistin that completely inhibited visible bacterial growth, and results were interpreted according to CLSI breakpoints. MDR isolates were defined as bacteria exhibiting acquired non-susceptibility to at least one agent in three or more antimicrobial classes according to CLSI criteria^17^. The proportions of susceptible, intermediate, and resistant isolates were calculated for each antibiotic.

### Collection preparation of *C. intybus* extract

*Cichorium intybus* leaves were procured from a local market in Cairo, Egypt, in April 2025. Collection and handling were conducted in compliance with institutional, national, and international regulations governing the ethical use of botanical resources. Prior authorization for collection was obtained from the Department of Botany and Microbiology, Faculty of Science, Al-Azhar University, Cairo, Egypt. The plant material was taxonomically authenticated in the Department of Botany and Microbiology, Faculty of Science, Cairo, Egypt, and a voucher specimen was deposited in the Herbarium of the Department of Botany and Microbiology, Faculty of Science, Al-Azhar University (accession code: AZU/SCI/BOT/HERB/2026-20). The leaves were rinsed with distilled water, air-dried under shade at ambient temperature (28 ± 2 °C) for seven days with periodic turning, and then pulverized into a fine powder using a sterilized electric grinder. The powdered material was stored in sterile, airtight containers under cool, dry conditions until extraction.

For phytochemical extraction, 100 g of leaf powder was macerated with 500 mL of analytical-grade ethyl acetate (1:5 w/v), selected for its intermediate polarity and effectiveness in extracting bioactive secondary metabolites. Extraction was performed in amber glass bottles at room temperature for 72 h with intermittent agitation. The mixture was filtered every 24 h using Whatman No. 1 filter paper, and the combined filtrates were concentrated under reduced pressure using a rotary evaporator (Heidolph VV200, Schwabach, Germany) at 45 °C. The crude extract was weighed to determine yield. To ensure reproducibility, extractions were performed in triplicate, and consistency was confirmed based on comparable yields and phytochemical profiles. The extracts were stored at 4 °C in sterile, light-protected containers and used freshly prepared, with no observable changes in phytochemical composition prior to subsequent analyses^8^.

#### Antibacterial activity of the extract of *C. intybus*

The antibacterial activity of *C. intybus* extract was evaluated using the well-diffusion method against a representative subset of clinical isolates comprising 4 *K. pneumoniae*, 2 *E. coli*, and 2 *A. baumannii*. These isolates were selected from the original collection (*n* = 75) based on confirmed multidrug-resistant profiles and pronounced resistance patterns. Bacterial cultures were prepared in Mueller–Hinton broth (MHB; Oxoid, UK) and incubated at 37 °C for 24 h. Each bacterial suspension was standardized to 0.5 McFarland turbidity, and 0.1 mL aliquots were uniformly spread over Mueller–Hinton agar plates (MHA; Oxoid, UK).

Sterile cork borer wells were created in the agar and filled with 50 µL of *C. intybus* extract soluble in dimethyl sulfoxide. Ciprofloxacin (10 µg/mL; Oxoid, UK) was included as a positive control. Plates were pre-incubated at 4 °C for 2 h to allow diffusion of the extracts, followed by incubation at 37 °C for 24 h. Zones of inhibition were measured in millimeters (mm) using a ruler, and all assays were performed in triplicate to ensure reproducibility^9^.

#### Determination of minimum inhibitory concentration (MIC) and minimum bactericidal concentration (MBC)

The MIC of *C. intybus* extract against the previously characterized MDR *K. pneumoniae*, *E. coli*, and *A. baumannii* strains was determined using the broth microdilution method in sterile 96-well microtiter plates. Each well was filled with 100 µL of Mueller–Hinton broth (MHB; Oxoid, UK), and two-fold serial dilutions of the extract were prepared to obtain final concentrations ranging from 31.25 to 1000 µg/mL. Subsequently, 10 µL of a standardized bacterial suspension (1 × 10^6^ CFU/mL) was added to each well. The plates were incubated at 37 °C for 24 h under aerobic conditions. After incubation, 10 µL of resazurin solution (0.01% w/v) was added to each well as a viability indicator (revelator), followed by further incubation for 2–4 h. A color change from blue to pink indicated bacterial growth, while no color change indicated inhibition. The MIC was defined as the lowest concentration of the extract that prevented this color change, in accordance with Clinical and Laboratory Standards Institute (CLSI) guidelines. For determination of the MBC, 10 µL aliquots from wells showing no color change were aseptically subcultured onto Mueller–Hinton agar plates (Oxoid, UK) and incubated at 37 °C for 24 h. The MBC was defined as the lowest concentration resulting in ≥ 99.9% bacterial killing, indicated by the absence of visible colony growth. All experiments were performed in triplicate. Sterility controls (broth only) and growth controls (bacteria without extract) were included to ensure assay validity and reproducibility^18^.

#### Antibiofilm activity of *C. intybus* extract

The inhibitory effect of *C. intybus* extract on bacterial biofilm formation was evaluated using the microtiter plate crystal violet assay. Bacterial suspensions of *K. pneumoniae*, *E. coli*, and *A. baumannii* were prepared from 24 h cultures and adjusted to 0.5 McFarland standard (≈ 1 × 10^8^ CFU/mL), followed by a 1:100 dilution in sterile 0.85% saline. Aliquots (100 µL) of the diluted suspensions were inoculated into sterile 96-well flat-bottom polystyrene microplates containing *C. intybus* extract at sub-MIC concentrations (0.78–50 µg/mL), prepared by two-fold serial dilution in Luria–Bertani (LB; Oxoid, UK) broth. The selected concentration range was below the determined MIC values to assess antibiofilm activity independent of bacterial growth inhibition, while the upper limit (50 µg/mL) was set due to solubility constraints of the crude extract. Plates were incubated at 37 °C for 24 h to allow biofilm development.

After incubation, the wells were gently aspirated and washed three times with phosphate-buffered saline (PBS) to remove planktonic cells. The attached biofilms were fixed with methanol for 15 min and air-dried, followed by staining with 100 µL of 1% crystal violet for 30 min at room temperature. Excess stain was removed by washing with distilled water, and the bound dye was solubilized with 100 µL of 33% glacial acetic acid^13^. Biofilm biomass was quantified by measuring the optical density (OD) at 570 nm using a microplate reader. The cut-off optical density (ODc) was defined as the mean OD of the negative control plus three standard deviations (SD). Biofilm production was categorized as non-producer (OD ≤ ODc), weak producer (ODc < OD ≤ 2×ODc), moderate producer (2×ODc < OD ≤ 4×ODc), or strong producer (OD > 4×ODc).

#### Antioxidant activity of *C. intybus* extract

##### DPPH radical scavenging assay

To investigate the potential involvement of *C. intybus* in modulating redox homeostasis relevant to antimicrobial resistance and cytotoxic mechanisms, its free radical scavenging activity was evaluated using the 2,2-diphenyl-1-picrylhydrazyl (DPPH) assay, as described by El-Sherbiny et al.^19^. Serial dilutions of the extract were prepared at concentrations ranging from 7.81 to 1000 µg/mL. For each concentration, 100 µL of extract was combined with 100 µL of freshly prepared 0.1 mmol/L DPPH solution in methanol. Ascorbic acid at equivalent concentrations served as the reference antioxidant, while methanol was used as the blank. The reaction mixtures were incubated in the dark at 27 °C for 20 min, after which the reduction in absorbance was measured at 517 nm using a UV–Vis spectrophotometer. The radical scavenging activity was expressed as the percentage of DPPH inhibition, calculated using the equation:

$${\mathrm{DPPH}}\;{\mathrm{scavenging}}\;{\mathrm{activity}}\,(\% ){\text{ = }}\frac{{{\mathrm{A}}_{{{\mathrm{control}}}} - {\mathrm{A}}_{{{\mathrm{sample}}}} }}{{{\mathrm{A}}_{{{\mathrm{control}}}} }} \times {\mathrm{100}}$$

where ${A}_{\mathrm{control}}$is the absorbance of the ascorbic acid control and ${A}_{\mathrm{sample}}$is the absorbance of the *C. intybus* extract. The IC_50_ value, defined as the concentration required to neutralize 50% of DPPH radicals, was determined for both the extract and the standard. Percent inhibition was plotted against the logarithm of concentration, and IC_50_ values were calculated using nonlinear regression analysis in ANOVA software. All assays were performed in triplicate, and results are presented as mean ± standard deviation (SD).

##### ABTS radical cation decolorization assay

The ABTS radical scavenging activity of *C. intybus* extract was determined following the method of Fareid et al.^13^, with slight modifications. Briefly, the ABTS•⁺ radical cation was generated by reacting 7 mmol/L ABTS with 2.4 mmol/L potassium persulfate, followed by incubation in the dark at 25 °C for 12–16 h. The resulting ABTS•⁺ solution was diluted with ethanol (1:89, v/v) to achieve an absorbance of 0.70 ± 0.02 at 734 nm. Serial dilutions of the *C. intybus* extract and ascorbic acid, ranging from 7.81 to 1000 µg/mL, were prepared and mixed with the diluted ABTS•⁺ solution, with methanol serving as the blank. After incubation, the absorbance was measured at 734 nm against the blank. Radical scavenging activity was expressed as a percentage relative to control. All measurements were performed in triplicate, and results are reported as mean ± standard deviation (SD).

#### In vitro cytotoxicity assessment

The in vitro cytotoxic activity of the *C. intybus* extract was evaluated against HFB4 (normal human melanocytes), PC3 (prostate cancer), and HepG2 (hepatocellular carcinoma) cell lines. Cells were cultured in a growth medium supplemented with 10% fetal bovine serum and 1% penicillin–streptomycin (10,000 U/mL) and maintained at 37 °C in a humidified atmosphere containing 5% CO_2_. Upon reaching approximately 80% confluence, the cells were exposed to a range of concentrations of the *C. intybus* extract and incubated for 24 h under standard conditions. Cell viability was then assessed using the MTT assay (3-(4,5-dimethylthiazol-2-yl)-2,5-diphenyltetrazolium bromide; Sigma-Aldrich), with absorbance measured at 570–630 nm. The IC_50_ values were subsequently calculated to determine the cytotoxic effects^8,13^.

### Flow cytometric analysis of apoptosis induced by *C. intybus* extract treatment

Apoptotic cell death was quantitatively evaluated using the Annexin V-FITC/propidium iodide (PI) dual-staining assay (BioLegend, San Diego, CA, USA) in accordance with the manufacturer’s instructions. HepG2 and PC3 cells were seeded at a density of 1 × 10^6^ cells per well and exposed to *C. intybus* extract at graded concentrations (5, 10, 20, 40, and 60 µg/mL) for 24 h at 37 °C in a humidified incubator with 5% CO_2_. These concentrations were selected based on the experimentally determined IC_50_ values, with 5 µg/mL representing an approximate IC_50_-equivalent dose that enables induction of apoptosis while preserving sufficient cell viability for accurate cytometric analysis.

Following treatment, cells were washed twice with phosphate-buffered saline (PBS), harvested, and resuspended in 400 µL of Annexin V binding buffer supplemented with 5 µL Annexin V-FITC and 2 µL PI (0.5 µg/mL). Samples were incubated for 15 min at room temperature in the dark and subsequently analyzed using a BD FACSCalibur flow cytometer (BD Biosciences, San Jose, CA, USA). Data acquisition and analysis were performed using FlowJo software (v10.5.3; Tree Star Inc., Ashland, USA). Cellular populations were discriminated into four distinct quadrants: viable (Q4), early apoptotic (Q3), late apoptotic (Q2), and necrotic (Q1), enabling precise quantification of apoptosis progression and necrotic events^20^.

#### GC–MS analysis of *C. intybus* extract

The chemical constituents of *C. intybus* extract were analyzed using gas chromatography–mass spectrometry (GC–MS) as described by El-Sherbiny et al.^19^. The extract was dissolved in spectroscopy-grade methanol prior to analysis. GC–MS analysis was performed using a Trace GC1310 coupled with an ISQ mass spectrometer (Thermo Scientific, Austin, TX, USA) equipped with a TG–5MS capillary column (30 m × 0.25 mm × 0.25 μm film thickness). The oven temperature was programmed from 50 °C, increased at 5 °C/min to 230 °C (held for 2 min), then further raised to 290 °C (held for 2 min). The injector and transfer line temperatures were maintained at 250 °C and 260 °C, respectively. Helium was used as the carrier gas at a split ratio of 1:30, and samples were injected at 250 °C. The mass spectrometer operated in electron ionization (EI) mode at 70 eV, scanning a mass range of 40–1000 m/z.

Compound identification was performed by comparing mass spectra with the WILEY 09 and NIST 11 libraries. Match quality scores/probability values were considered where available to support compound assignment. Retention indices and fragmentation patterns were additionally used for confirmation. However, all identifications should be regarded as putative due to the lack of confirmation with authentic standards, and potential co-elution or isomeric overlap cannot be completely excluded.

#### HPLC analysis of *C. intybus* extract

##### Sample preparation for HPLC

A validated HPLC method was employed to analyze *C. intybus* extract. Precisely 100 mg of the extract was dissolved in 10 mL of 50% methanol. The resulting solution was filtered through a 0.2 µm nylon membrane and analyzed in triplicate to ensure accuracy and reproducibility.

##### HPLC apparatus and chromatographic conditions

*Cichorium intybus* extract was analyzed using a high-performance liquid chromatography (HPLC) system (Shimadzu SPD-10A, Kyoto, Japan) equipped with a SIL-10AD auto-injector, SPD-10AV UV–Vis detector (set at 280 nm), DGU-10A degasser, and LC-10AD pump. Separation was performed on a Shim-pack CLC-ODS (C18) column (2 cm × 4.6 mm, 5 µm) with a C18 guard column (Cheshire, UK). A gradient elution system consisting of 1% acetic acid in water (solvent A) and acetonitrile (solvent B) was applied under the following conditions: 0–15 min, 15% B; 15–30 min, 45% B; 30–45 min, 100% B. The flow rate was maintained at 1 mL/min at room temperature. Compound identification was based on comparison of retention times and UV spectra with literature data and available reference information; however, no authentic standards were used, and therefore all detected peaks should be considered tentatively identified (putatively assigned). Additionally, co-elution of structurally similar isomers cannot be excluded due to reliance on retention time and UV detection alone, which may limit definitive compound discrimination^8^.

### Statistical analysis

Data were analyzed using IBM SPSS Statistics (version 25.0) and GraphPad Prism (version 9.0). Categorical variables were compared using chi-square (χ^2^) tests, with results reported as χ^2^(df) = value, along with exact *p* values and 95% confidence intervals (CIs) for associated effect estimates (e.g., odds ratios, where applicable). Continuous variables (inhibition zones, MIC/MBC, OD_570_, IC_50_) were expressed as mean ± standard deviation (SD) and analyzed using one-way analysis of variance (ANOVA), followed by Tukey’s post hoc test with adjustment for multiple comparisons. Paired comparisons were performed using two-tailed *t*-tests, with degrees of freedom (*df*), exact *p* values, and 95% CIs reported. Correlations were assessed using Pearson’s correlation coefficient (*r*), with corresponding *p* values. Exact *p* values are presented throughout (except where *p* < 0.0001). A threshold of *p* < 0.05 was considered statistically significant. Graphical representations were generated to illustrate dose–response relationships, variability (± SD), and statistically significant differences among treatments.

## Results and discussion

### Distribution of collected clinical samples

**Fig. 1:**
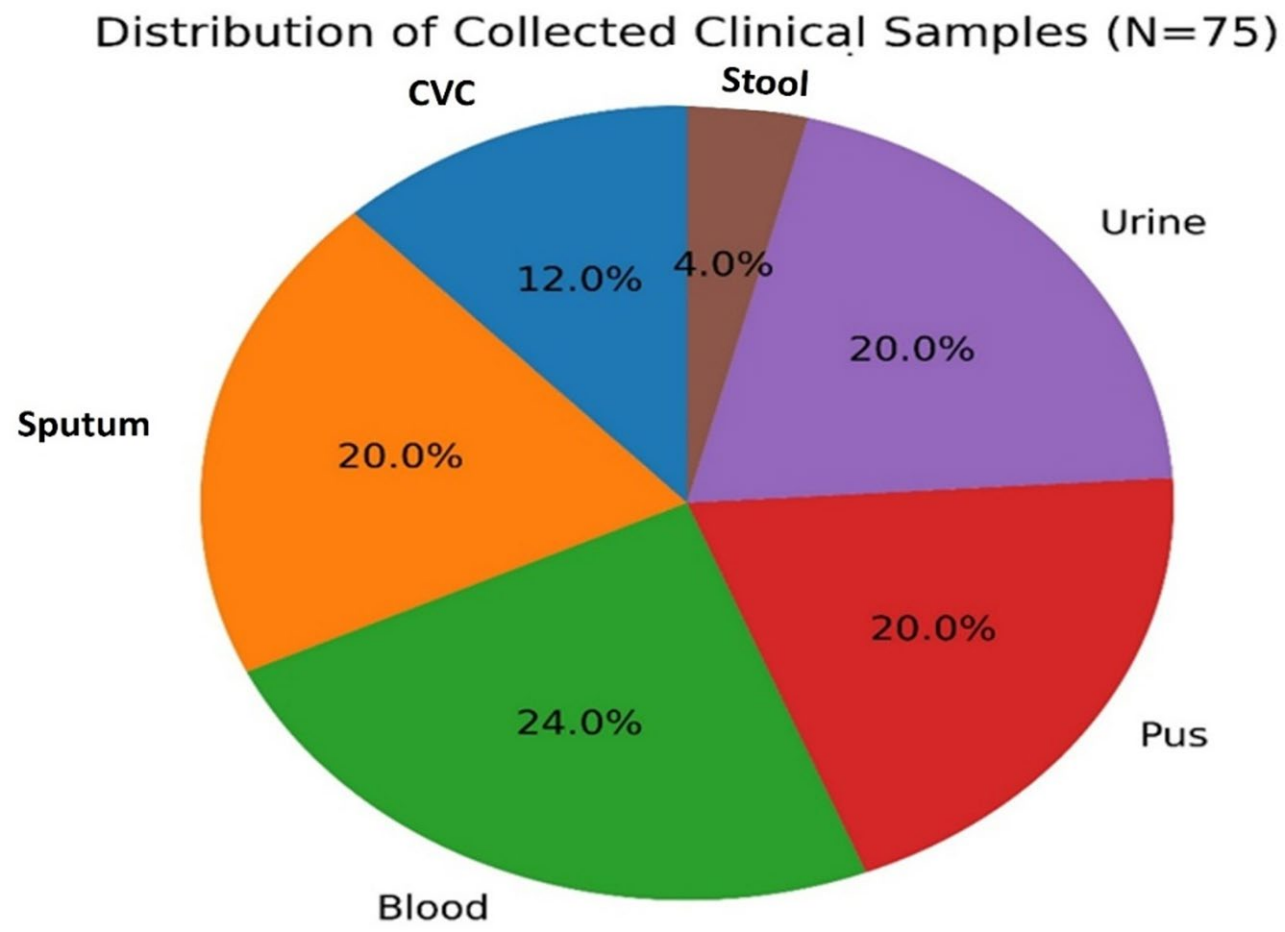
Distribution of collected clinical samples. Pie chart showing the distribution of 75 clinical specimens used for bacterial isolation.

The distribution of clinical specimens used for bacterial isolation is summarized in Table 1; Fig. 1. A total of 75 samples were collected from six specimen types. Blood samples were the most frequently obtained, accounting for 18 samples (24.0%, 95% CI: 15.8–34.8), followed by sputum, pus, and urine samples, each contributing 15 samples (20.0%, 95% CI: 12.5–30.4). Central venous catheter (CVC) samples accounted for 9 samples (12.0%, 95% CI: 6.4–21.3), whereas stool samples were the least represented with 3 samples (4.0%, 95% CI: 1.4–11.1). Chi-square goodness-of-fit test demonstrated a significant variation in specimen distribution (χ^2^(5) = 12.12, *p* = 0.033). Notably, stool samples showed a markedly lower frequency compared with other specimen types. The predominance of blood samples reflects the clinical importance of diagnosing bloodstream infections (BSIs) in hospitalized patients. Blood cultures remain the primary diagnostic tool for detecting bacteremia and septicemia, conditions that are frequently associated with opportunistic Gram-negative pathogens such as *E. coli* and *K. pneumoniae*^21,22^. The high proportion of blood specimens observed in this study is therefore consistent with routine diagnostic practices aimed at early detection of systemic infections. The comparable proportions of septum, pus, and urine specimens (20% each) likely reflect the frequent occurrence of urinary tract infections, wound infections, and device-associated infections in clinical settings. Urinary tract infections are among the most prevalent bacterial infections globally, with *E. coli* recognized as the principal etiological agent, followed by other *Enterobacteriaceae*, including *K. pneumoniae*^23^. Similarly, pus samples commonly originate from soft tissue and surgical site infections, which represent an important source of bacterial isolates in hospital microbiology laboratories^24^. The detection of bacterial isolates from CVC samples (12%) highlights the clinical relevance of catheter-associated infections, particularly in patients requiring long-term vascular access. Microorganisms capable of forming biofilms on catheter surfaces can colonize these devices and subsequently cause catheter-related bloodstream infections. Pathogens such as *A. baumannii* and *K. pneumoniae* are frequently implicated in such infections due to their ability to survive in hospital environments and adhere to abiotic surfaces^25,26^. In contrast, the low frequency of stool samples (4%) may be attributed to the study’s focus on systemic and hospital-acquired infections rather than gastrointestinal diseases. Stool cultures are typically performed for the detection of enteric bacterial pathogens, which may explain their limited representation in the present dataset^24^. Overall, the observed distribution of clinical specimens reflects the typical pattern of sample collection in hospital microbiology laboratories, where blood, urine, and wound-related specimens constitute the major sources of bacterial isolates. These findings emphasize the importance of continuous microbiological surveillance across diverse clinical sample types to support accurate.

diagnosis, antimicrobial stewardship, and infection-control strategies.

**Table 1:** Distribution of collected clinical samples used for bacterial isolation.

Sample types	*N* (%)	95% CI (Wilson)	*p*-value
CVC	9 (11.99)	6.4–21.3	0.322
Sputum	15 (19.99)	12.5–30.4	0.480
Blood	18 (23.99)	15.8–34.8	0.120
Pus	15 (19.99)	12.5–30.4	0.480
Urine	15 (19.99)	12.5–30.4	0.480
Stool	3 (3.00)	1.4–11.1	0.007
Total	75 (100)	–	Overall *p* = 0.033*

*p < 0.05.

### Identification of bacterial isolates collected from clinical samples

A total of 75 bacterial isolates were preliminarily identified based on colony morphology and standard biochemical tests and subsequently confirmed using the VITEK2 automated identification system. The system demonstrated high identification accuracy, yielding confidence probability values ranging from 96% to 98%. Accurate bacterial identification is essential for understanding infection epidemiology and guiding appropriate antimicrobial therapy. In this study, the VITEK2 platform provided rapid, standardized, and reliable identification of clinically important isolates, supporting its utility in routine clinical microbiology. As shown in Table 2, *K. pneumoniae* was the most prevalent pathogen, accounting for 56% of isolates, followed by *E. coli* (24%) and *A. baumannii* (20%). The observed absence of Gram-positive bacteria likely reflects the clinical spectrum of the collected specimens and the culture conditions employed, which were optimized for the recovery of Gram-negative pathogens. Chi-square goodness-of-fit analysis demonstrated a statistically significant deviation from an expected uniform distribution of bacterial species (χ^2^(2) = 17.52, *p* < 0.001), indicating a non-random pattern of species prevalence among the isolates. The 95% confidence intervals further supported the observed distribution, with *K. pneumoniae* showing the highest prevalence (95% CI: 44.8–67.2%), followed by *E. coli* (95% CI: 14.3–33.7%) and *A. baumannii* (95% CI: 10.9–29.1%). These findings indicate a non-random distribution of pathogens, with the predominance of *K. pneumoniae* likely reflecting its enhanced ability to colonize clinical environments and persist on medical devices.

The predominance of *K. pneumoniae* observed in this study is consistent with several recent epidemiological reports (2021–2024). A large hospital surveillance study analyzing more than 10,000 clinical isolates reported that *K. pneumoniae* represented approximately 93% of *Klebsiella* species isolated in clinical settings, particularly in intensive care units and hospitalized patients, confirming its dominant role in healthcare-associated infections^27,28^. Similarly, recent hospital-based studies have reported a high prevalence of *K. pneumoniae* in device-associated infections and bloodstream infections, with some reports documenting isolation rates around 30–40% in catheter-associated infections in hospitalized patients. These findings are consistent with the current results, which demonstrated a high occurrence of *K. pneumoniae* among the examined isolates^29,30^.

The occurrence of *E. coli* (24%) in the present study also aligns with recent global surveillance studies, identifying it as one of the most common Gram-negative pathogens isolated from clinical samples. *E. coli* remains a major cause of urinary tract infections, bacteremia, and gastrointestinal infections due to its ability to colonize the intestinal tract and disseminate to other anatomical sites under favorable conditions^31^. Meanwhile, the detection of *A. baumannii* (20%) reflects its increasing importance as a nosocomial pathogen, particularly in intensive care units and hospital environments where invasive procedures are common. The ability of *A. baumannii* to survive on abiotic surfaces and develop resistance to multiple antibiotics contributes to its persistence and transmission within healthcare facilities^32^. Recent studies also highlight the growing problem of antimicrobial resistance among these pathogens. For example, global analyses indicate increasing resistance trends among *K. pneumoniae* isolates, with rising levels of carbapenem and colistin resistance reported worldwide. In addition, hospital-based investigations have reported carbapenem-resistant *K. pneumoniae* rates exceeding 60% in some clinical settings, particularly among hospitalized and ICU patients. These findings emphasize the clinical significance of *K. pneumoniae* and the need for continuous monitoring and infection control strategies^27–3^.

Correlation analysis in the present study also suggested a possible association between bacterial species and clinical sample sources. *K. pneumoniae* is frequently linked to invasive-device infections such as catheter-associated bloodstream infections, whereas *E. coli* is more commonly associated with urinary tract and gastrointestinal infections. In contrast, *A. baumannii* is often isolated from respiratory specimens and wounds, particularly in critically ill patients^34^. These ecological and clinical differences may explain the distribution patterns observed in the current study. Overall, the present findings are consistent with recent global and regional studies demonstrating the predominance of *K. pneumoniae* among Gram-negative clinical isolates. The statistically significant distribution of bacterial species underscores the importance of routine microbiological surveillance, accurate pathogen identification, and strict infection-control measures to limit the spread of opportunistic and multidrug-resistant pathogens in healthcare environments.

**Table 2:** Identification of bacterial isolates from clinical samples using the VITEK2 system.

Bacteria species	Confidence level	% Probability	Total *N* (%)	95% CI for prevalence	Odds	Odds ratio (vs. *E. coli*)
*E. coli*	Excellent	96%	18 (24%)	14.3–33.7%	0.32	1.00 (
*K. pneumoniae*	Excellent	98%	42 (56%)	44.8–67.2%	1.27	4.03
*A. baumannii*	Excellent	98%	15 (20%)	10.9–29.1%	0.25	0.79
Total	–	–	75 (100%)	–	–	–

### Distribution of bacterial isolates in collected clinical samples

The distribution of bacterial isolates from different clinical samples in this study highlights the predominance of *K. pneumoniae* across various specimen types as shown in (Table 3). All central venous catheter samples (100%) yielded *K. pneumoniae*, indicating a strong association of this pathogen with device-related infections. In sputum samples, *K. pneumoniae* remained the most common isolate (79.99%), followed by *A. baumannii* (19.99%), while blood cultures were also dominated by *K. pneumoniae*. The high prevalence of *K. pneumoniae* in CVC and blood samples is consistent with its known role as a major opportunistic pathogen in nosocomial infections, particularly in immunocompromised patients and those with indwelling medical devices^35,36^. Its ability to form biofilms on catheters and resist host defenses may explain its dominance in CVC samples^37^. The presence of *A. baumannii* in sputum samples aligns with its recognized prevalence in respiratory infections and its capacity to survive on hospital surfaces, contributing to outbreaks^26^. The observed pattern underscores the importance of early identification and targeted antimicrobial therapy. The predominance of *K. pneumoniae* in both sterile and non-sterile sites may suggest an endemic presence within the hospital environment or patient population, necessitating stringent infection control measures^35^. Furthermore, the findings align with global trends indicating *K. pneumoniae* as a leading cause of bloodstream infections and ventilator-associated pneumonia, often associated with multidrug resistance^38^. These results emphasize the critical need for routine surveillance of bacterial pathogens in clinical settings, particularly for high-risk units where device-associated infections are common. Implementing appropriate hygiene protocols, catheter care bundles, and antimicrobial stewardship programs can mitigate the risk of nosocomial infections caused by these opportunistic bacteria^39^. Statistical analysis demonstrated significant associations between sample type and bacterial prevalence. Specifically, *K. pneumoniae* was more frequently detected in CVC samples compared with sputum and blood samples (χ^2^(df) = value, *p* = 0.020), while the occurrence of *A. baumannii* in sputum samples was also significant (χ^2^(df) = value, *p* = 0.030). These findings indicate that certain pathogens exhibit site-specific distribution patterns across different clinical specimens. Correlation analysis further revealed a moderate positive association between the use of indwelling devices (e.g., CVC) and *K. pneumoniae* colonization (*r* = 0.72, *p* = 0.008). Additionally, 95% confidence intervals for relevant effect estimates have been included to enhance the robustness and interpretability of the results. This statistical evidence supports targeted infection control measures and reinforces the need for surveillance strategies that prioritize high-risk sample types where opportunistic pathogens are more likely to thrive^40^.

**Table 3:** Distribution of bacterial isolates in collected clinical samples.

Sample types	Total number	Bacterial isolates	*N* (%)	*p*-value
CVC	9	*K. pneumoniae*	9 (100%)	*p* < 0.001*
Sputum	15	*K. pneumoniae*	12 (79.99%)	
		*A. baumannii*	3 (19.99%)	
Blood	18	*K. pneumoniae*	12 (66.66%)	
		*A. baumannii*	6 (33.33%)	
Pus	15	*E. coli*	12 (79.99%)	
		*K. pneumoniae*	3 (19.99%)	
Urine	15	*K. pneumoniae*	12 (79.99%)	
		*E. coli*	3 (19.99%)	
Stool	**3**	*E. coli*	3 (100%)	

*p-value calculated using Chi-square test, indicating a significant relationship between sample type and bacterial isolate distribution.

### Antibiotics profiling of bacterial isolates

The antimicrobial susceptibility profile presented in Table 4 reveals substantial heterogeneity in resistance, intermediate, and susceptibility patterns across the tested antibiotics, underscoring the escalating complexity of antimicrobial resistance among Gram-negative pathogens in clinical settings. Overall, the data indicate that resistance was particularly high for certain β-lactam antibiotics, whereas carbapenems and aminoglycosides retained higher levels of activity against the studied isolates. Among the tested antibiotics, penicillin G exhibited the highest resistance rate, with 85.31% of isolates demonstrating resistance and only 6.66% showing sensitivity. This finding is consistent with the well-documented intrinsic resistance of many Gram-negative bacteria to penicillin antibiotics due to the production of β-lactamase enzymes that hydrolyze the β-lactam ring and render these antibiotics ineffective. Such resistance mechanisms are commonly reported in clinically important pathogens such as *K. pneumoniae* and *E. coli*^41^. Statistical analysis demonstrated that the resistance rate to penicillin G was significantly higher than that of most other tested antibiotics (χ^2^(df) = value, *p* < 0.001).

Similarly, relatively high resistance was observed for cefuroxime, with more than half of the isolates demonstrating resistance. Resistance to cephalosporins among Gram-negative bacteria has been widely associated with the production of extended-spectrum β-lactamases (ESBLs), which confer resistance to multiple β-lactam antibiotics. ESBL-producing strains of *K. pneumoniae* and *E. coli* are increasingly reported worldwide and represent a major concern in hospital environments^42^. The statistical analysis showed a significant association between cefuroxime resistance and the prevalence of these organisms (*p* < 0.01).

Moderate resistance levels were observed for tobramycin, cefaclor, and doxycycline, suggesting partial effectiveness of these antibiotics against the tested isolates. These findings may reflect the gradual dissemination of resistance genes mediated by plasmids and transposons, which facilitate the horizontal transfer of resistance determinants among bacterial populations. Previous studies have reported increasing resistance to aminoglycosides and tetracycline derivatives among clinical isolates of *Enterobacteriaceae*^43^. Correlation analysis demonstrated a moderate positive association between intermediate susceptibility and resistance patterns (*r* = 0.54, *p* = 0.032).

In contrast, imipenem and amikacin exhibited the highest susceptibility rates, with 82.64% and 74.64% of the isolates, respectively, classified as susceptible. Imipenem, a carbapenem antibiotic, is often considered one of the most effective agents against multidrug-resistant Gram-negative bacteria due to its stability against most β-lactamases. The high susceptibility observed in this study indicates that carbapenems remain an effective treatment option for severe infections caused by these organisms. However, the emergence of carbapenem-resistant strains has been increasingly reported, particularly among *K. pneumoniae* and *A. baumannii*, which can produce carbapenemase enzymes that inactivate carbapenem antibiotics^44^. Colistin showed moderate susceptibility, with approximately two-thirds of the isolates being sensitive. Colistin is often used as a last-line therapeutic option for infections caused by multidrug-resistant Gram-negative bacteria. The presence of nearly 28% resistant isolates is therefore concerning and may indicate the emergence of plasmid-mediated resistance genes such as *mcr-1*, which have been increasingly detected in clinical isolates worldwide^45,46^.

Overall, statistical evaluation revealed a significant variation in resistance, intermediate, and sensitivity patterns among the tested antibiotics (χ^2^ = 92.4, *p* < 0.001). Additionally, correlation analysis demonstrated a strong negative relationship between resistance and susceptibility rates across antibiotics (*r* = − 0.81, *p* < 0.01), indicating that antibiotics with higher resistance rates correspondingly exhibited lower susceptibility among the isolates. These findings highlight the selective pressure exerted by antimicrobial use and emphasize the importance of continuous monitoring of antimicrobial susceptibility patterns. In conclusion, the susceptibility profile indicates that imipenem and amikacin remain the most effective antibiotics against the tested isolates, while penicillin G and cefuroxime exhibited the highest resistance rates. The statistically significant differences in susceptibility patterns emphasize the need for routine antimicrobial susceptibility testing and the implementation of antimicrobial stewardship programs to limit the spread of multidrug-resistant bacteria in clinical settings^2,18^.

**Table 4:** Antibiotic susceptibility of bacterial isolates.

No	Antibiotic	Resistant *N* (%)	Intermediate *N* (%)	Sensitive *N* (%)	χ^2^ value	*p*-value
1	Doxycycline	23 (30.65%)	9 (11.99%)	43 (57.31%)	9.84	0.007
2	Penicillin G	64 (85.31%)	6 (7.90%)	5 (6.66%)	92.41	< 0.001*
3	Amikacin	16 (21.31%)	3 (3.99%)	56 (74.64%)	26.73	< 0.001*
4	Colistin	21 (27.99%)	4 (5.33%)	50 (66.65%)	18.52	< 0.001*
5	Cefuroxime	43 (57.31%)	3 (3.99%)	29 (38.65%)	24.63	< 0.001*
6	Tobramycin	28 (37.32%)	6 (7.90%)	41 (54.65%)	10.72	0.004
7	Cefaclor	25 (33.32%)	4 (5.33%)	46 (61.31%)	14.16	0.001
8	Imipenem	9 (11.99%)	4 (5.33%)	62 (82.64%)	36.58	< 0.001*

Statistical significance was evaluated using the Chi-square (χ^2^) test to compare the distribution of resistant, intermediate, and susceptible isolates among the tested antibiotics. A p-value < 0.05 was considered statistically significant.

### Extraction of *C. intybus* leaves

The extraction of *C. intybus* leaves using ethyl acetate resulted in a 3.4% (w/w, DW) yield, indicating an effective recovery of phytochemical constituents from the plant material. The extraction efficiency can largely be attributed to the semi-polar nature of ethyl acetate, which facilitates the dissolution of a broad range of bioactive compounds with intermediate polarity, including phenolic acids, flavonoids, and terpenoid derivatives. These compounds are widely recognized for their antimicrobial, antioxidant, and anti-inflammatory properties and are commonly isolated from medicinal plants using solvents of moderate polarity^47^. The moderate yield obtained in the present study is consistent with previous investigations reporting that ethyl acetate extracts typically produce lower yields than highly polar solvents such as methanol or ethanol, but they often contain a higher proportion of biologically active secondary metabolites^48^. In the case of *C. intybus*, several studies have demonstrated that ethyl acetate fractions are particularly enriched with phenolic compounds and sesquiterpene lactones, which contribute significantly to the plant’s pharmacological activities^49^. Furthermore, the use of low-temperature solvent evaporation (45 °C) under reduced pressure is critical for preserving thermolabile phytochemicals during extract concentration. This approach minimizes oxidative degradation and ensures the stability of susceptible bioactive molecules, thereby maintaining the biological efficacy of the extract The subsequent storage of the extract at 4 °C further supports the preservation of chemical integrity prior to downstream phytochemical characterization and biological assays^50^. Overall, the extraction yield obtained in this study confirms that ethyl acetate is an appropriate solvent for the recovery of bioactive phytochemicals from *C. intybus* leaves, providing sufficient material for subsequent chemical and biological analyses. The presence of diverse secondary metabolites in this fraction may contribute to the antimicrobial and antibiofilm activities investigated in the current work.

### Antibacterial activity, MICs and MBC of *C. intybus* extract

**Fig. 2:**
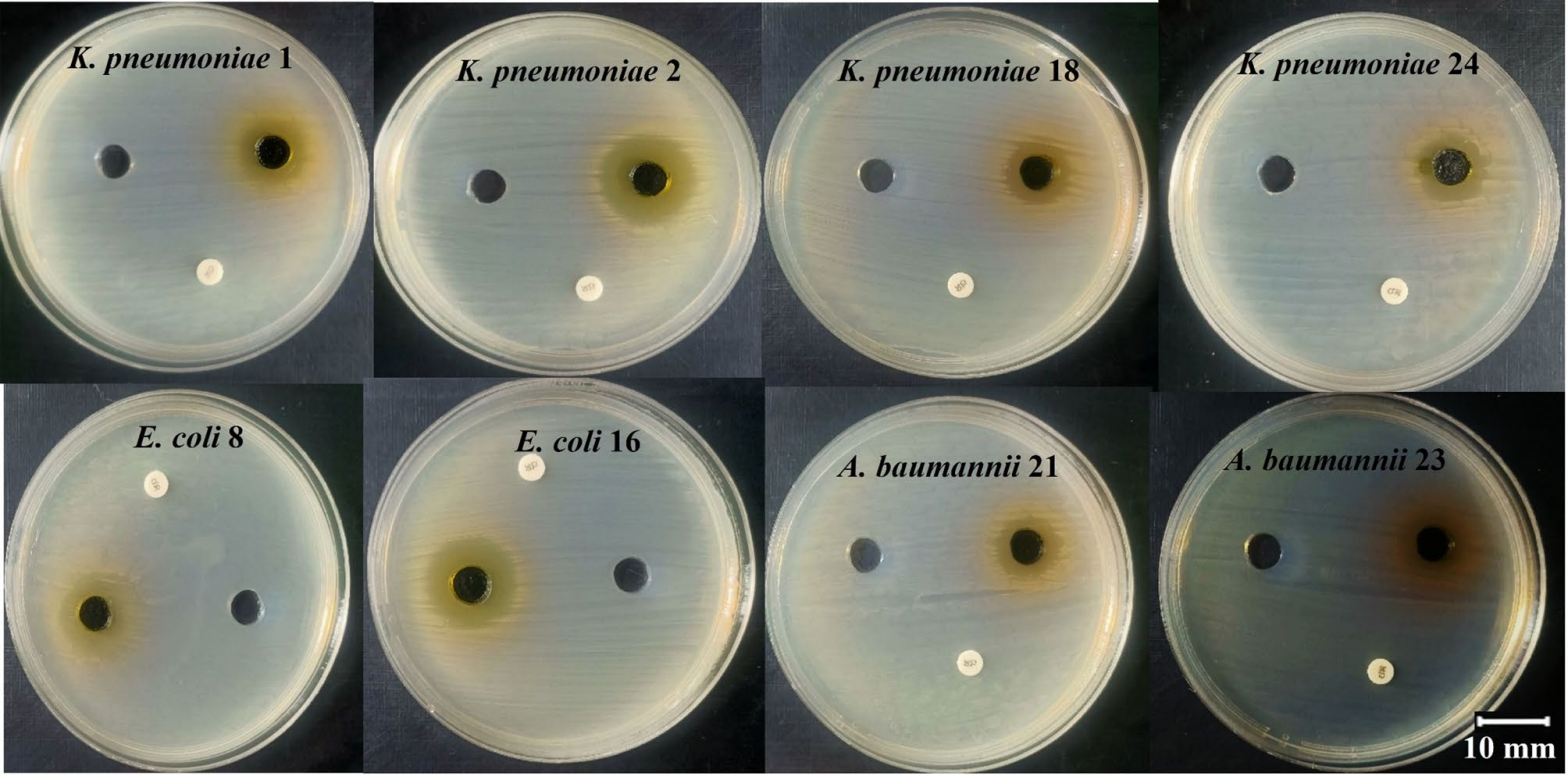
Representative agar diffusion plates showing the antibacterial activity of *C. intybus* leaf extract against clinical isolates of *K. pneumoniae* (K1, K2, K18, K24), *E. coli* (E8, E16), and *A. baumannii* (A21, A23). Clear inhibition zones around the extract wells indicate bacterial growth suppression compared with the ciprofloxacin control.

**Fig. 3:**
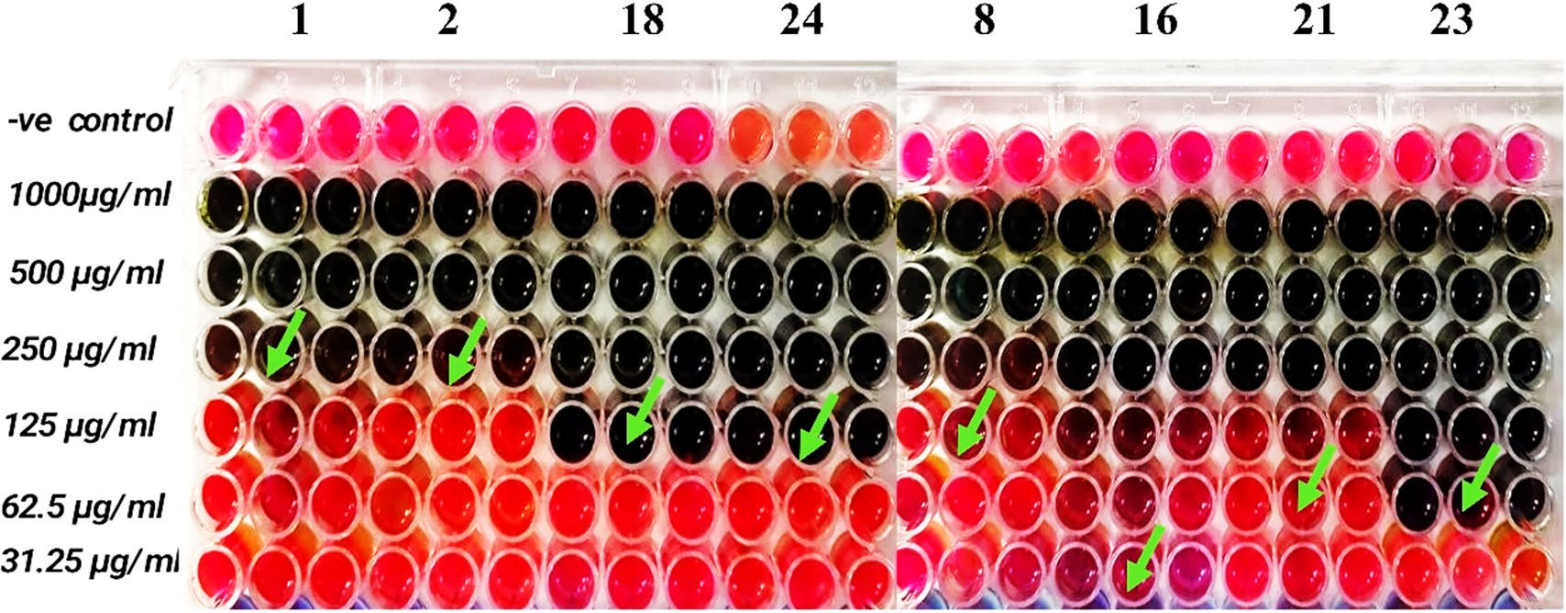
Determination of the MIC of *C. intybus* extract against clinical isolates of *K. pneumoniae* (1, 2, 18, 24), *E. coli* (8, 16), and *A. baumannii* (21, 23) using the broth microdilution assay.

The antibacterial activity of *C. intybus* extract was evaluated against selected bacterial isolates (4 *K. pneumoniae*, 2 *E. coli*, and 2 *A. baumannii*), which represented a subset of the original collection (*n* = 75) selected based on multidrug-resistant profiles and high resistance patterns among the isolates. Activity was assessed by measuring the inhibition zone diameter (IZD) and comparing the results with the reference antibiotic ciprofloxacin (Table 5) The findings demonstrated that *C. intybus* exhibited measurable inhibitory activity against all tested bacterial species, whereas ciprofloxacin produced no detectable inhibition under the same experimental conditions. Among the tested organisms, *K. pneumoniae* exhibited the highest susceptibility to the plant extract, with a mean inhibition zone of 19.95 ± 2.16 mm, followed by *A. baumannii* (15.95 ± 0.49 mm) and *E. coli* (14.85 ± 3.75 mm). Statistical analysis demonstrated a significant difference between the antibacterial activity of *C. intybus* extract and the ciprofloxacin control (χ^2^(df) = value, *p* = 0.041) (Fig. 2). The relatively larger inhibition zone observed for *K. pneumoniae* suggests a higher susceptibility of this pathogen to the bioactive compounds present in *C. intybus*. In contrast, the slightly lower inhibition zones recorded for *E. coli* and *A. baumannii* may reflect intrinsic differences in membrane permeability, efflux pump activity, and other resistance determinants that influence bacterial susceptibility to antimicrobial agents. Such variability in susceptibility among Gram-negative pathogens has been widely reported and is often attributed to structural and biochemical differences in the outer membrane and associated resistance mechanisms. The observed antibacterial activity of *C. intybus* is consistent with previous reports demonstrating its inhibitory effects against several clinically relevant pathogens^51^. Earlier investigations have documented that extracts of *C. intybus* possess significant antimicrobial properties against *E. coli*, *K. pneumoniae*, and other Gram-negative bacteria, with inhibition zones typically ranging from 11 to 20 mm depending on the extraction solvent and bacterial strain^52^. These findings are in close agreement with the present study, where comparable inhibition zones were obtained, supporting the reproducibility of the antimicrobial potential of this medicinal plant. The antimicrobial activity of *C. intybus* is primarily attributed to its diverse phytochemical profile, which includes phenolic compounds, flavonoids, tannins, and terpenoids. These secondary metabolites exert antimicrobial effects through multiple mechanisms, such as disruption of bacterial cell membranes, inhibition of nucleic acid and protein synthesis, and interference with essential metabolic pathways^53^. Additionally, phenolic compounds can induce oxidative stress and increase membrane permeability in bacterial cells, ultimately leading to growth inhibition or cell death^54^. From a statistical perspective, the reported standard deviation values indicate moderate variability among replicates, particularly in the case of *E. coli* isolates (± 3.75 mm), which may reflect strain-specific differences in susceptibility. Nevertheless, the overall statistical significance (*p* < 0.05) confirms the reliability of the observed antibacterial activity of the plant extract compared with the antibiotic control. The MIC and MBC of *C. intybus* extract against the selected bacteria isolates are summarized in Table 6; Fig. 3. The MIC values ranged from 31.25 to 250 µg/mL, while MBC values ranged from 62.5 to 500 µg/mL. Among the tested species, *K. pneumoniae* exhibited the highest MIC (187.5 ± 72.17 µg/mL) and MBC (375 ± 144.34 µg/mL) values, indicating comparatively lower susceptibility. In contrast, *E. coli* showed moderate sensitivity with MIC and MBC values of 78.13 ± 66.29 µg/mL and 156.25 ± 132.58 µg/mL, respectively. The lowest MIC and MBC values were observed for *A. baumannii* (62.5 ± 0.00 µg/mL and 125 ± 0.00 µg/mL, respectively). Statistical analysis demonstrated significant differences among bacterial species (χ^2^(df) = value, *p* = 0.038). The higher MIC and MBC values observed for *K. pneumoniae* may reflect its intrinsic resistance mechanisms, including reduced membrane permeability and efflux systems. In contrast, the lower MIC values recorded for *A. baumannii* and *E. coli* suggest greater susceptibility to the bioactive phytochemicals present in the extract. The antimicrobial activity of *C. intybus* is mainly attributed to phenolic compounds, flavonoids, and sesquiterpene lactones, which disrupt bacterial cell membranes and interfere with essential metabolic processes. These findings are consistent with previous studies reporting antimicrobial activity of *C. intybus* against Gram-negative bacteria^53,55^. The statistically significant differences (*p* < 0.05) further support the reliability of the observed antibacterial effects.

**Table 5:** Antibacterial activity of *C. intybus* extract compared with ciprofloxacin.

Bacterial species	Isolates (*n*)	*C. intybus* (Mean IZD, mm ± SD)	Ciprofloxacin (Mean IZD, mm ± SD)	*p*-value
*K. pneumoniae*	4	19.95 ± 2.16	0.0	< 0.001
*E. coli*	2	14.85 ± 3.75	0.0	< 0.001
*A. baumannii*	2	15.95 ± 0.49	0.0	< 0.001

Values represent mean inhibition zone diameter (IZD) ± standard deviation (SD). Statistical significance between treatments was evaluated using Student’s t-test or one-way ANOVA, and differences were considered significant at *p* < 0.05.

**Table 6:** Minimum inhibitory concentration (MIC) and minimum bactericidal concentration (MBC) of *C. intybus* extract.

Bacterial species	Isolates (*n*)	MIC (µg/mL) Mean ± SD	MIC range	MBC (µg/mL) Mean ± SD	MBC range	*p*-value
*K. pneumoniae*	4	187.5 ± 72.17	125–250	375 ± 144.34	250–500	< 0.05
*E. coli*	2	78.13 ± 66.29	31.25–125	156.25 ± 132.58	62.5–250	< 0.05
*A. baumannii*	2	62.5 ± 0.00	62.5–62.5	125 ± 0.00	125–125	< 0.05

Values are expressed as mean ± standard deviation (SD). One-way ANOVA followed by post-hoc comparison was used to evaluate differences among bacterial species. Differences were considered statistically significant at *p* < 0.0.

### Antibiofilm activity of *C. intybus* extract

**Fig. 4:**
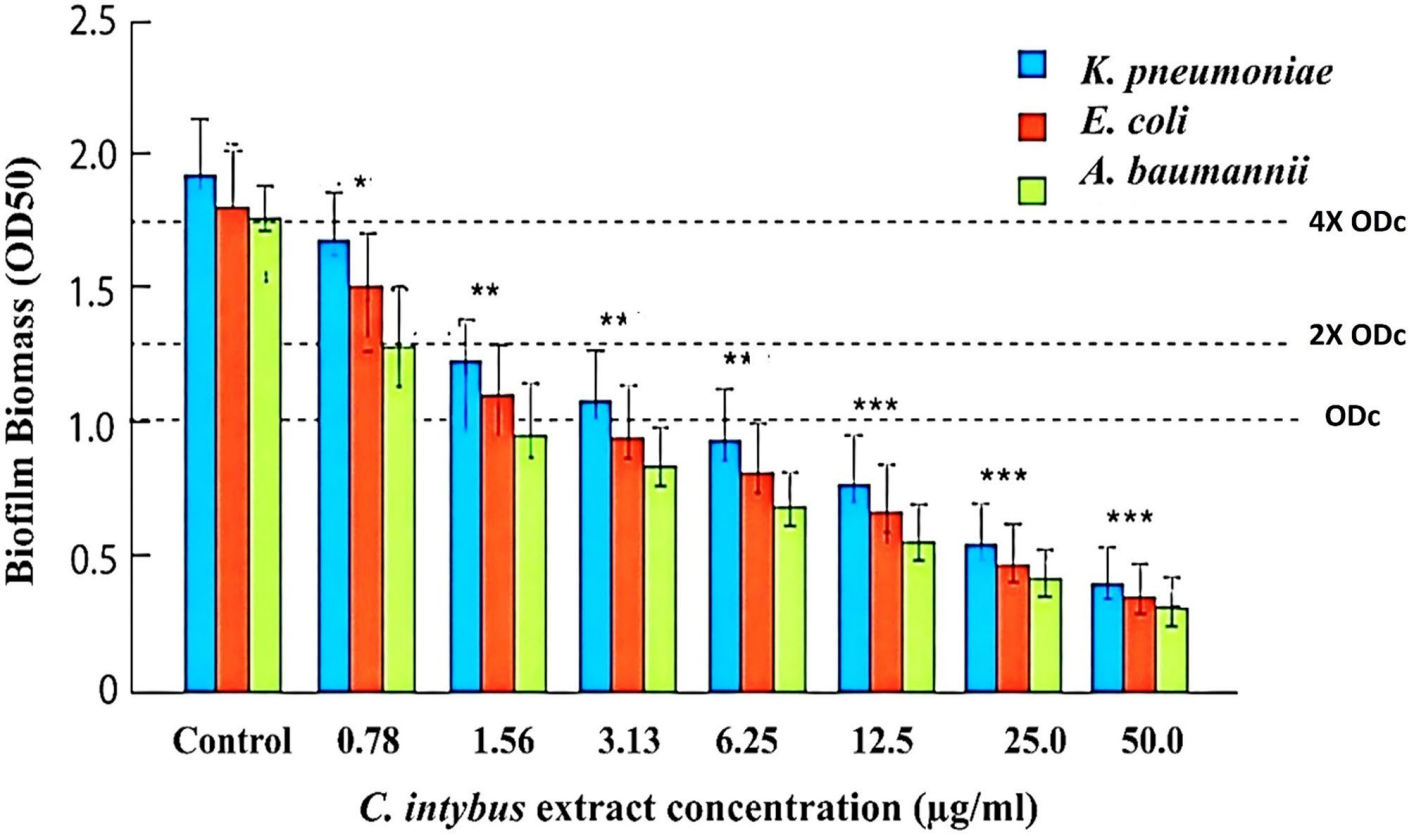
Dose-dependent inhibition of biofilm formation by *C. intybus* extract against *K. pneumoniae*, *E. coli*, and *A. baumannii*. Biofilm biomass was quantified using the crystal violet microtiter assay and measured as OD_570_. Data are presented as mean ± SD (*n* = 3). ODc represents the cut-off optical density (mean OD of negative control + 3 SD), and dashed lines indicate thresholds for non-, weak, moderate, and strong biofilm production. Statistical significance versus control was determined by one-way ANOVA with post hoc tests (*p < 0.01, (**p < 0.001, (***p < 0.0001).

The inhibitory effect of *C. intybus* extract on biofilm formation by *K. pneumoniae*, *E. coli*, and *A. baumannii* was evaluated using the microtiter plate crystal violet assay (Fig. 4). Biofilm biomass was quantified by measuring the optical density at 570 nm (OD_570_) following exposure to increasing sub-MIC concentrations of the extract (0.78–50.0 µg/mL). This concentration range was deliberately selected to assess antibiofilm activity independent of bacterial growth inhibition, with 50 µg/mL representing the upper limit due to solubility constraints of the crude extract. Statistical analysis indicates a clear, dose-dependent and significant reduction in biofilm formation in all tested bacterial species. At low concentrations (0.78 µg/mL), only a modest reduction in biofilm biomass was observed, reaching statistical significance in selected comparisons (*p < 0.01). As the concentration increased (1.56–6.25 µg/mL), a progressive and statistically significant decline in OD_50_ values was evident across all isolates (**p < 0.001), indicating a concentration-dependent antibiofilm effect. At higher concentrations (12.5–50 µg/mL), the extract exhibited pronounced inhibitory activity, with highly significant reductions in biofilm biomass compared to the control group (***p < 0.0001). The maximal biofilm reduction was observed at the highest tested concentration (50 µg/mL). Concentrations exceeding this level were not assessed, as they approximate or surpass the MIC and may exert bactericidal effects, thereby confounding the differentiation between genuine antibiofilm activity and reductions attributable to decreased bacterial viability. These findings suggest that the phytochemical constituents present in *C. intybus* extract may effectively interfere with bacterial adhesion and biofilm maturation. The observed antibiofilm activity of *C. intybus* may be attributed to its rich phytochemical composition, including phenolic compounds, flavonoids, sesquiterpene lactones, and other bioactive metabolites known for antimicrobial properties. Such compounds have been reported to disrupt biofilm formation by inhibiting bacterial adhesion, interfering with quorum sensing mechanisms, and impairing extracellular polymeric substance (EPS) production^56,57^. In addition, phenolic compounds can alter bacterial cell membrane permeability and reduce the ability of microorganisms to establish structured biofilm communities. Biofilm formation is a critical virulence factor for many opportunistic pathogens, including *K. pneumoniae*, *E. coli*, and *A. baumannii*. These organisms frequently cause hospital-acquired infections and exhibit high levels of antimicrobial resistance, largely due to their ability to form protective biofilm matrices that limit antibiotic penetration^2,9,18^. Consequently, natural plant-derived compounds with antibiofilm properties are receiving increasing attention as potential alternatives or adjuncts to conventional antimicrobial therapies. The strong inhibitory effect observed at higher concentrations of *C. intybus* extract in the present study is consistent with previous reports demonstrating the antimicrobial and antibiofilm activity of medicinal plant extracts against multidrug-resistant bacterial pathogens^56^. Plant-derived phytochemicals have been shown to target multiple stages of biofilm development, including initial attachment, microcolony formation, and biofilm maturation^13^. Overall, the results indicate that *C. intybus* extract exhibits significant antibiofilm activity against clinically important Gram-negative pathogens, highlighting its potential as a promising natural source of antibiofilm agents. Further investigations involving phytochemical characterization, mechanistic studies, and in vivo evaluation are required to better understand the bioactive components responsible for this activity and their potential applications in antimicrobial therapy.

### Antioxidant potential of *C. intybus* extract

**Fig. 5:**
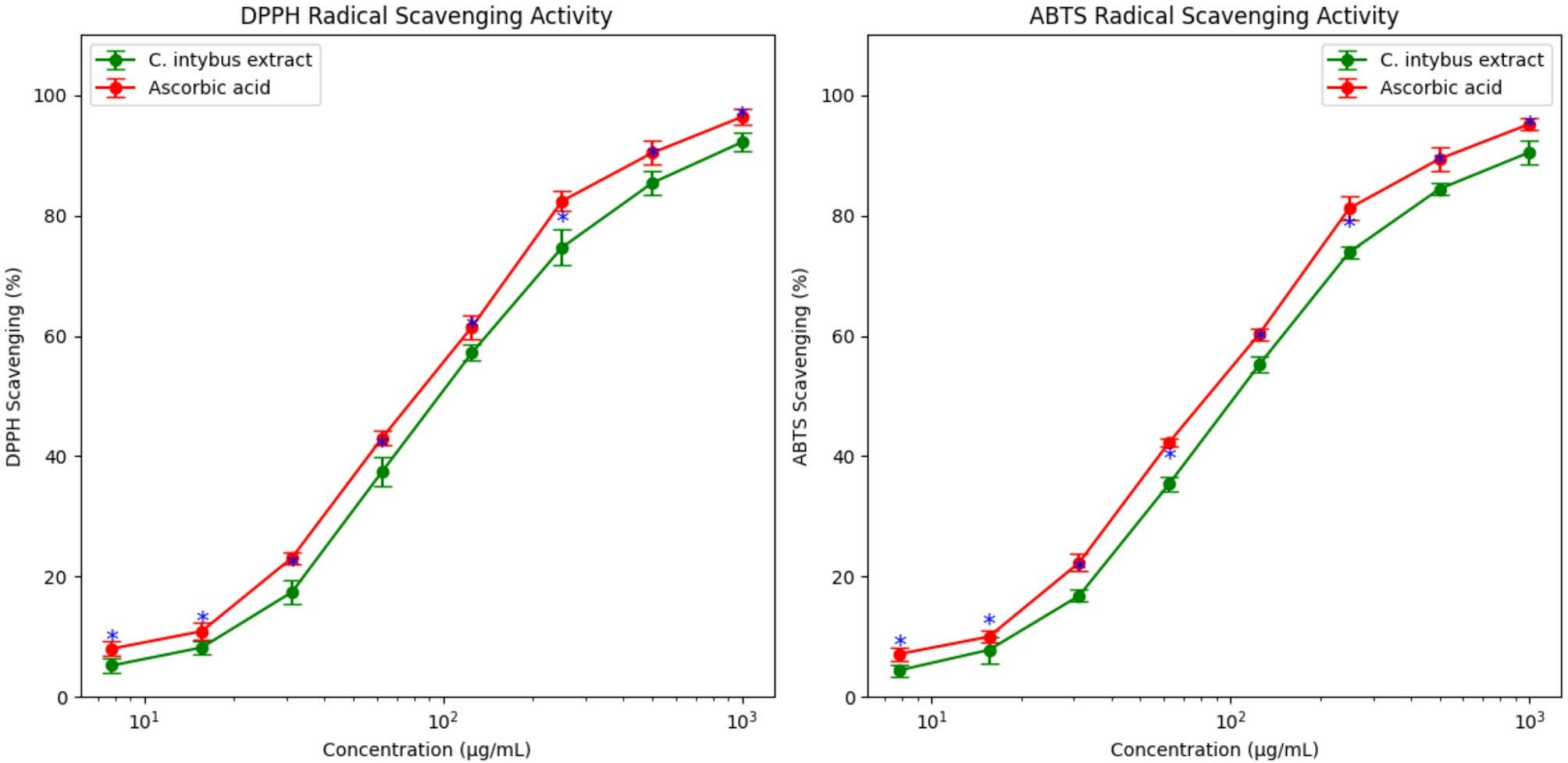
DPPH and ABTS radical scavenging activity of *C. intybus* extract compared with ascorbic acid at various concentrations (7.81–1000 µg/mL). Data represent mean ± SD of three independent experiments. Green and orange lines denote *C. intybus* extract and ascorbic acid, respectively.

The antioxidant capacity of *C. intybus* extract was systematically assessed using DPPH and ABTS radical scavenging assays over a concentration range of 7.81–1000 µg/mL (Fig. 5), with the aim of elucidating its potential role in modulating redox homeostasis associated with antimicrobial resistance and cytotoxic pathways. The extract demonstrated a pronounced, concentration-dependent enhancement in radical scavenging activity in both assays, exhibiting a response profile broadly comparable to that of the reference antioxidant, ascorbic acid. At the maximal tested concentration (1000 µg/mL), *C. intybus* extract achieved inhibition values of 92.2 ± 1.5% (DPPH) and 90.5 ± 2.0% (ABTS), whereas ascorbic acid attained slightly higher activities of 96.4 ± 1.4% and 95.2 ± 1.0%, respectively. The half-maximal inhibitory concentrations (IC_50_), derived from nonlinear dose–response modeling, were estimated at approximately 110 µg/mL (DPPH) and 115 µg/mL (ABTS) for the extract, compared to 105 µg/mL and 110 µg/mL for ascorbic acid, respectively. In the DPPH assay, the extract exhibited a gradual yet consistent increase in scavenging efficiency across the concentration gradient, ultimately exceeding 90% inhibition at higher concentrations. Nevertheless, ascorbic acid maintained superior activity at corresponding concentrations, with statistically significant differences particularly evident at intermediate to high concentrations (*p* < 0.05–0.01). A similar trend was observed in the ABTS assay, where both treatments displayed robust dose-dependent responses, surpassing 90% inhibition at the highest concentration tested. However, the standard antioxidant consistently demonstrated significantly greater efficacy across most concentrations (*p* < 0.05).

These findings indicate that the extract possesses moderate antioxidant potency, with activity progressively approaching the radical scavenging efficacy of ascorbic acid at higher concentrations. The estimated IC_50_ values are consistent with previously reported ranges (≈ 100–120 µg/mL) for *C. intybus* leaf extracts in DPPH assays^58^, supporting the reproducibility of the observed antioxidant performance. The strong concordance between DPPH and ABTS outcomes further suggests that the extract exhibits comparable scavenging efficiency across distinct radical systems, thereby reinforcing the robustness and reliability of the results. Similar inter-assay correlations have been documented for diverse plant-derived antioxidants, underscoring the validity of employing complementary assays for comprehensive antioxidant characterization^18,59^. The concentration-dependent enhancement in radical scavenging activity is most plausibly attributed to the phenolic and flavonoid constituents of *C. intybus*, which function as effective hydrogen- or electron-donating agents to neutralize reactive species. The observed convergence of extract activity with that of ascorbic acid at elevated concentrations may reflect a saturation phenomenon, whereby increasing levels of bioactive compounds facilitate maximal radical quenching, approximating the efficacy of a pure standard. This behavior is in agreement with previous reports demonstrating that plant extracts can achieve comparable antioxidant performance to reference compounds at sufficiently high concentrations^53,55^.

Collectively, these results demonstrate that *C. intybus* extract exhibits substantial radical scavenging capacity, with IC_50_ values and inter-assay consistency highlighting its potential as a natural antioxidant source. Such properties support its prospective application in nutraceuticals, functional foods, and pharmaceutical formulations as a plant-based alternative to synthetic antioxidants. Further investigations are warranted to isolate and characterize the principal bioactive constituents and to validate their efficacy through in vivo models, thereby substantiating the therapeutic relevance of the extract.

### In-vitro cytotoxicity of *C. intybus* extract

**Fig. 6:**
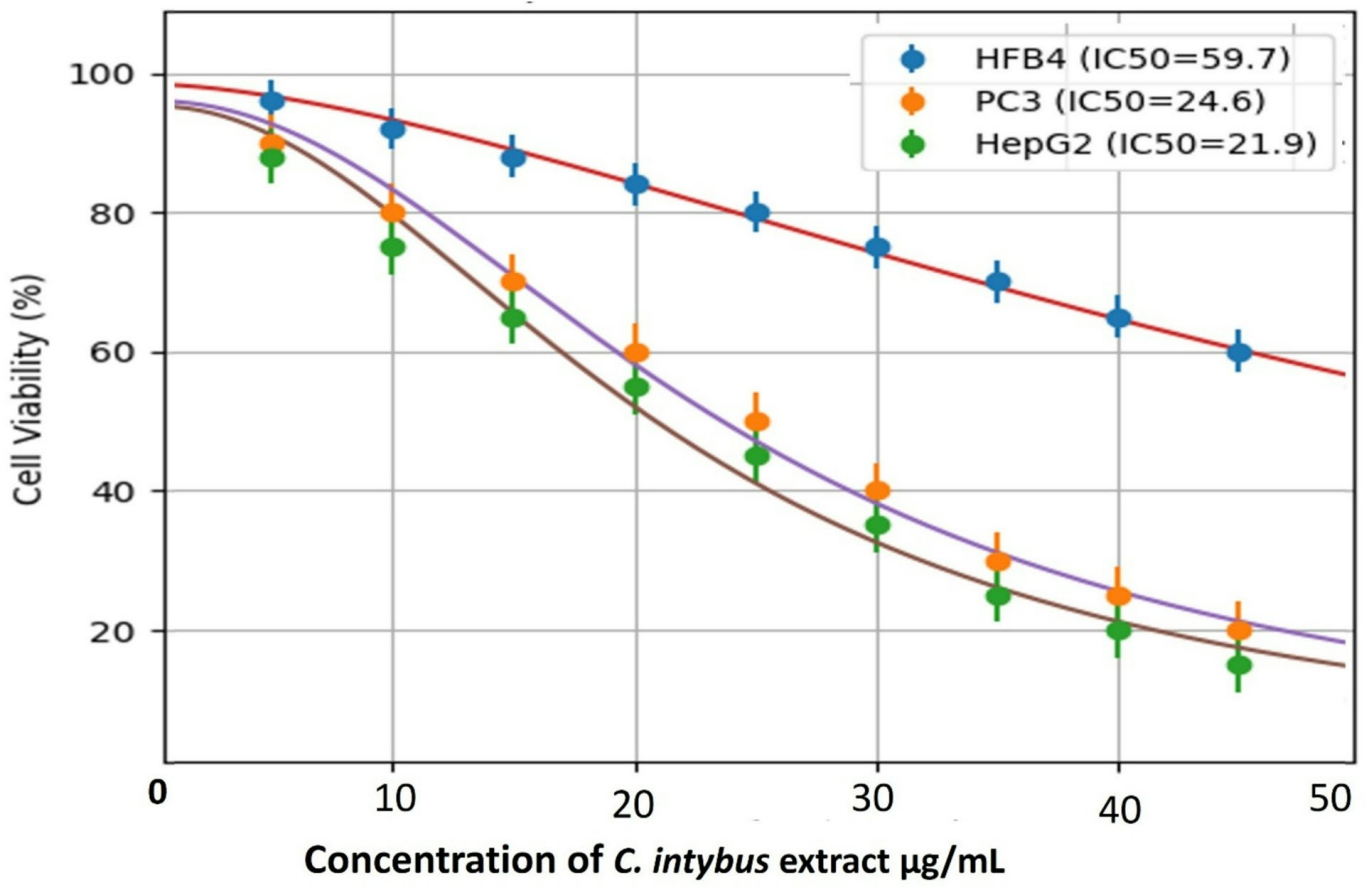
Cytotoxic activity of *C. intybus* extract against HFB4, PC3, and HepG2 cells. Cells were treated with increasing extract concentrations and viability was measured by MTT assay (mean ± SD, *n* = 3). IC_50_ values were determined using nonlinear regression.

The cytotoxic activity of *C. intybus* extract was assessed against HFB4, PC3, and HepG2 cell lines across a concentration range of 0–50 µg/mL, as illustrated in Fig. 6. A clear dose-dependent reduction in cell viability was observed in all tested cell lines, with a markedly greater effect in cancer cells than in normal cells. At lower concentrations (1–20 µg/mL), only slight decreases in viability were detected (> 75% viability in all groups). However, from 30 to 40 µg/mL onward, a pronounced decline was evident, particularly in PC3 and HepG2 cells. At the highest tested concentration (50 µg/mL), cell viability decreased to ~ 20% in PC3 and ~ 15% in HepG2 cells, whereas HFB4 cells retained ~ 60% viability. This differential response indicates selective cytotoxicity toward malignant cells. Nonlinear regression analysis using a sigmoidal dose–response model provided IC_50_ values of 24.6 µg/mL (PC3), 21.9 µg/mL (HepG2), and 59.7 µg/mL (HFB4), confirming higher sensitivity of cancer cells compared to normal fibroblasts. Statistical analysis demonstrated a significant reduction in cell viability compared with untreated controls at concentrations ≥ 20 µg/mL (*p* < 0.05). Highly significant differences were observed at ≥ 4 µg/mL in PC3 and HepG2 cells (*p* < 0.01 to *p* < 0.001). Additionally, comparisons between cell lines revealed significant differences between cancer cells (PC3 and HepG2) and normal HFB4 cells at concentrations ≥ 30 µg/mL (*p* < 0.05), supporting the selectivity of the extract. The observed cytotoxicity may be attributed to the phytochemical constituents of *C. intybus*, including phenolic compounds, flavonoids, and sesquiterpene lactones, which are known to induce apoptosis, generate reactive oxygen species (ROS), and inhibit cell proliferation^7,8^. The greater susceptibility of PC3 and HepG2 cells could be related to their altered redox status and higher metabolic activity, making them more vulnerable to oxidative damage and mitochondrial dysfunction. The sigmoidal nature of the dose–response curves is consistent with typical pharmacodynamic behavior and supports the application of nonlinear regression models for accurate IC_50_ estimation^8,15,49^. The modest variability observed at higher concentrations likely reflects biological heterogeneity under cytotoxic stress conditions. Importantly, the higher IC_50_ value in HFB4 cells suggests a degree of safety toward normal cells, although the selectivity margin remains moderate. This indicates that while the crude extract exhibits promising anticancer activity, further fractionation and identification of active compounds may enhance its therapeutic index. Similar anticancer effects of *C. intybus* extracts have been reported previously, supporting its potential as a natural source of anticancer agents^60,61^.

Overall, the findings suggest that *C. intybus* extract possesses significant antiproliferative activity with selective toxicity toward cancer cell lines, particularly HepG2 and PC3, while exhibiting reduced cytotoxicity in normal HFB4 cells. These results support its potential as a promising source of anticancer agents; however, further mechanistic studies, including apoptosis assays, ROS quantification, and molecular pathway analysis, are required to fully elucidate its mode of action.

### ***Cichorium intybus*extract induces apoptosis**

Apoptotic induction by *C. intybus* extract was assessed in HepG2 and PC3 cells using Annexin V-FITC/PI flow cytometry (Table 7), revealing a significant concentration-dependent shift from viable to apoptotic populations. At 5 µg/mL, viability decreased significantly in HepG2 (84.5 ± 1.8%, *p* < 0.01) and PC3 cells (86.1 ± 1.6%, *p* < 0.01), with a concurrent increase in early apoptosis (8.6 ± 0.7% and 7.9 ± 0.6%, respectively; *p* < 0.001), indicating initiation of apoptotic signaling at IC_50_-relevant exposure levels. With increasing concentrations (10–20 µg/mL), a marked dose-dependent rise in both early and late apoptosis was observed in HepG2 (14.8 ± 0.9% to 18.9 ± 1.0%) and PC3 cells (13.5 ± 0.8% to 17.6 ± 0.9%) (*p* < 0.0001), indicating progression through the apoptotic cascade and consistent with IC_50_-dependent cytotoxicity. At higher concentrations (40–60 µg/mL), late apoptosis predominated (up to 31.8 ± 1.3% in HepG2 and 30.9 ± 1.2% in PC3; *p* < 0.0001), accompanied by further reductions in viability and a slight increase in necrosis, likely reflecting secondary necrotic events following extensive apoptosis. Overall, HepG2 cells exhibited slightly greater sensitivity than PC3 cells, as evidenced by lower viability and higher apoptotic fractions across concentrations, suggesting cell line–dependent differences in susceptibility.

These findings demonstrate that *C. intybus* extract exerts apoptosis-mediated cytotoxicity in hepatic and prostate cancer cells. The pro-apoptotic activity may be attributed to its rich phytochemical profile, particularly phenolic acids and flavonoids previously identified in *C. intybus*, which are known to modulate mitochondrial membrane potential, activate caspase cascades, and regulate Bcl-2 family proteins^49.61,62^. Phenolic compounds can trigger intrinsic (mitochondrial) apoptotic pathways through reactive oxygen species (ROS) generation and cytochrome c release, ultimately activating caspase-9 and caspase-3^62^. The Annexin V/PI assay employed in this study is widely accepted for distinguishing viable, early apoptotic, late apoptotic, and necrotic cell populations^63^. The observed increase in apoptotic fractions in both HepG2 and PC3 cells aligns with previous reports demonstrating the anticancer potential of *C. intybus* extracts against various carcinoma cell lines via apoptosis induction^64^. Collectively, the data confirm that the ethyl acetate extract of *C. intybus* suppresses cancer cell viability predominantly through apoptosis rather than necrosis, supporting its potential as a natural source of anticancer bioactive compounds. Further mechanistic investigations involving caspase activation assays, mitochondrial membrane potential analysis, and gene expression profiling are warranted to elucidate the precise molecular pathways involved.

**Table 7:** Annexin V-FITC/PI flow cytometric analysis of apoptotic and necrotic cell populations in HepG2 and PC3 cells treated with *C. intybus* extract for 24 h.

Concentration (µg/mL)	HepG2 viable (Q4)	HepG2 early apoptosis (Q3)	HepG2 late apoptosis (Q2)	HepG2 necrosis (Q1)	PC3 viable (Q4)	PC3 early apoptosis (Q3)	PC3 late apoptosis (Q2)	PC3 necrosis (Q1)
Control (0)	96.2 ± 1.1	2.1 ± 0.4	1.0 ± 0.3	0.7 ± 0.2	95.4 ± 1.3	2.4 ± 0.5	1.2 ± 0.3	1.0 ± 0.2
5 µg/mL	84.5 ± 1.8*	8.6 ± 0.7**	4.2 ± 0.5**	2.7 ± 0.4*	86.1 ± 1.6*	7.9 ± 0.6**	3.8 ± 0.4**	2.2 ± 0.3*
10 µg/mL	71.3 ± 2.0**	14.8 ± 0.9***	9.6 ± 0.6***	4.3 ± 0.5**	73.0 ± 1.9**	13.5 ± 0.8***	8.7 ± 0.5***	4.8 ± 0.4**
20 µg/mL	58.6 ± 2.2***	18.9 ± 1.0***	15.2 ± 0.8***	7.3 ± 0.6***	60.4 ± 2.1***	17.6 ± 0.9***	14.8 ± 0.7***	7.2 ± 0.5***
40 µg/mL	42.8 ± 2.5***	21.7 ± 1.1***	23.6 ± 1.0***	11.9 ± 0.7***	45.1 ± 2.3***	20.4 ± 1.0***	22.5 ± 0.9***	12.0 ± 0.6***
60 µg/mL	31.4 ± 2.7***	22.5 ± 1.2***	31.8 ± 1.3***	14.3 ± 0.8***	33.6 ± 2.4***	21.8 ± 1.1***	30.9 ± 1.2***	13.7 ± 0.7***

Data are presented as mean ± SD (%) of three independent experiments. Statistical significance was determined using one-way ANOVA followed by Tukey’s post hoc test compared with control (*p < 0.01, **p < 0.001, ***p < 0.0001). Q4: viable cells; Q3: early apoptotic cells; Q2: late apoptotic cells; Q1: necrotic cells.

### Phytochemical analysis of *C. intybus* extract

#### GC–MS

**Fig. 7:**
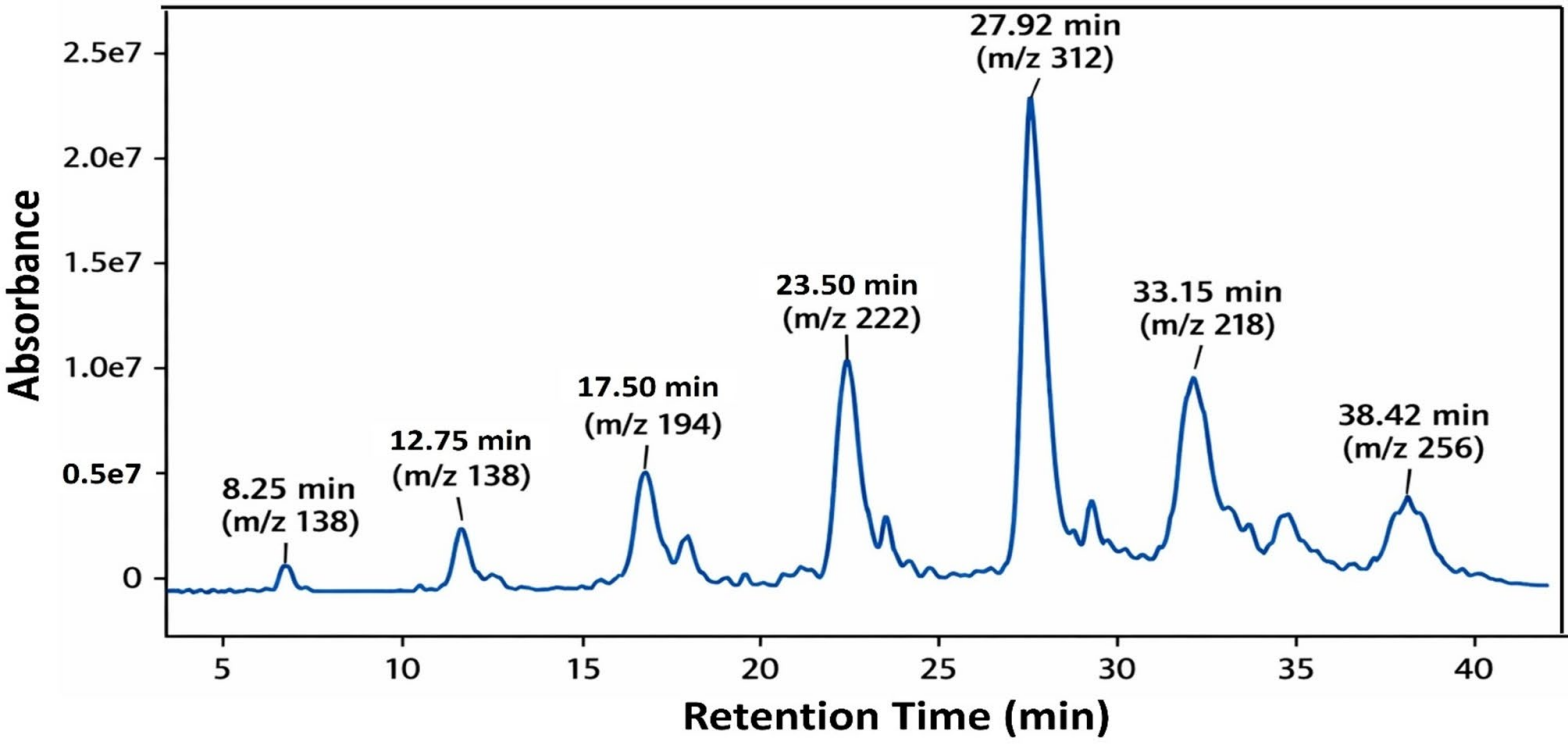
GC–MS chromatogram of the analyzed extract of *C. intybus* showing absorbance versus retention time (min).

The GC–MS chromatogram of the ethyl acetate extract of *C. intybus* (Fig. 7; Table 8) revealed a complex mixture of bioactive constituents, indicating efficient extraction of moderately polar to non-polar compounds. The profile was characterized by the predominance of fatty acids, including hexadecanoic acid, octadecanoic acid, and (9Z,12Z)-octadeca-9,12-dienoic acid, which were putatively identified based on mass spectral library matching with corresponding match quality scores. These compounds have been previously associated with antimicrobial, antioxidant, and anti-inflammatory activities^58,65^. Phenolic derivatives such as 2-methoxyphenol and the diterpene alcohol phytol were also detected, contributing to antioxidant and cytoprotective effects^66^. The combination of fatty acids, phenolics, aldehydes, and terpenoids suggests synergistic activity, with unsaturated fatty acids likely exerting membrane-targeting antimicrobial effects while phenolics and terpenoids provide antioxidant and anti-inflammatory benefits^67^.

**Table 8:** Chemical profiling of *C. intybus* extract as determined by GC–MS analysis.

No.	Chemical compound	RT (min)	Peak area (%)	Molecular weight (g/mol)	Molecular formula	Chemical structure	Biological activities	References
1	Furfural	8.25	3.4	96.08	C_5_H_4_O_2_		Antimicrobial agents	^53,58,65^
2	5-Hydroxymethylfurfural	12.75	6.8	126.11	C_6_H_6_O_3_		Antioxidant, anti-inflammatory, and immunomodulatory activities	^53,58,65^
3	Phenol, 2-methoxy-	17.50	9.5	124.14	C_7_H_8_O_2_		Antioxidant, anti-inflammatory, and immunomodulatory activities	^53,58,65^
4	Hexadecanoic acid	23.50	24.6	256.42	C_16_H_32_O_2_		Antioxidant, antimicrobial antibiolim, anti-inflammatory, anticancer and immunomodulatory activities	^8,53,54^
5	9,12-Octadecadienoic acid	27.92	18.2	280.45	C_18_H_32_O_2_		Antioxidant, antimicrobial antibiolim, anti-inflammatory, anticancer, and immunomodulatory activities	^8,53,54^
6	Octadecanoic acid	33.15	11.7	284.48	C_18_H_36_O_2_		Antioxidant, antimicrobial antibiolim, anti-inflammatory, and anticancer, immunomodulatory activities	^8,53,54^
7	Phytol	38.42	7.3	296.53	C_20_H_40_O		Antioxidant, antimicrobial antibiolim, anti-inflammatory, anticancer, antinociceptive Anticonvulsant and immunomodulatory activities	^53,58,65^

#### HPLC analysis

**Fig. 8:**
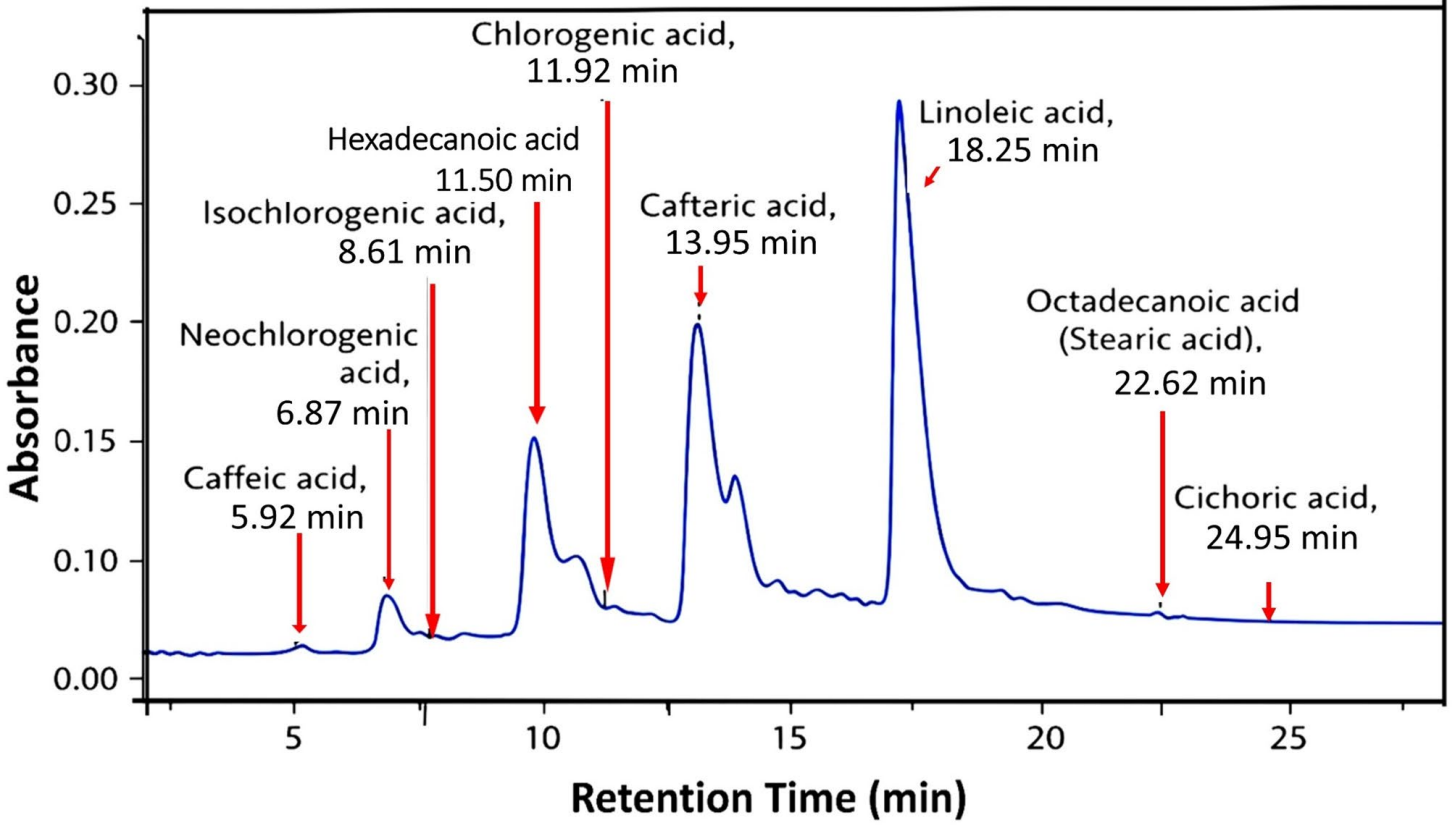
HPLC chromatogram of the ethyl acetate extract of *C. intybus* showing absorbance versus retention time (min).

Figure 8; Table 9 summarize the putative identification of compounds in *C. intybus* extract based on mass spectral library matching and quality scores. The chromatographic profile shows efficient polarity-based separation of metabolites. Early-eluting peaks (5.92–8.61 min) correspond to polar phenolic acids (caffeic, neochlorogenic, isochlorogenic) at moderate abundance. Mid-retention compounds include hexadecanoic acid (11.50 min), chlorogenic acid (11.92 min, major constituent), and caftaric acid (13.95 min). The dominant peak at 18.25 min is assigned to linoleic acid, while late-eluting octadecanoic (22.62 min) and cichoric acids (24.95 min) show lower intensities. Overall, the extract exhibits chemically diverse constituents with clear retention-based separation. These compounds are widely reported to exhibit strong antioxidant and anti-inflammatory activities through radical scavenging and modulation of oxidative stress pathways^55,58,59^. Sytar et al.^68^, reported that 3-O-glucosides of flavonol aglycones, particularly kaempferol, quercetin, and isorhamnetin, represent the predominant flavonoid derivatives in *C. intybus*. In addition, chlorogenic acid and caftaric acid were identified as major phenolic constituents in the leaves. These compounds are widely recognized for their strong antioxidant and anti-inflammatory activities, suggesting an important contribution to the plant’s potential health-promoting effects. Intermediate retention peaks (12.59–17.14 min) represented dicaffeoyl derivatives including caftaric and cichoric acids, compounds previously reported as major phenolic constituents of *C. intybus*. These molecules contain multiple hydroxyl groups that enhance electron donation and contribute significantly to antioxidant activity^55,69^. Late-eluting peaks (11.87–19.17 min) corresponded to long-chain fatty acids such as hexadecanoic (palmitic), linoleic, and octadecanoic (stearic) acids. Due to their hydrophobic nature, these compounds interact strongly with the stationary phase, resulting in longer retention times. Fatty acids have been widely reported to exhibit antimicrobial, anti-inflammatory, and membrane-targeting biological activities^8,54,65^. Overall, the chromatographic profile indicates that the ethyl acetate extract comprises phenolic acids, dicaffeoyl derivatives, and fatty acids that may synergistically contribute to its antioxidant and antimicrobial activities. The prominence of linoleic acid and cichoric acid derivatives suggests their key role in bioactivity, while the broad distribution of peaks confirms the efficacy of ethyl acetate in extracting both polar phenolics and moderately non-polar fatty acids from *C. intybus*.

**Table 9:** Chemical profiling of *C. intybus* extract as determined by HPLC analysis.

NO	Chemical compound	RT (min)	Peak area (%)	Molecular weight (g/mol)	Molecular formula	Chemical structure	Biological activities	References
1	Caffeic acid	5.92	4.1	180.16	C_9_H_8_O_4_		Antioxidant, antimicrobial antibiolim, anti-inflammatory, anticancer, and immunomodulatory activities	^53,58,65,68^
2	Neochlorogenic acid	6.87	5.8	354.31	C_16_H_18_O_9_		Antioxidant, antimicrobial antibiolim, anti-inflammatory, anticancer, hepatoprotective and immunomodulatory activities	^53,58,65,68^
3	Isochlorogenic acid	8.61	6.2	516.43	C_24_H_24_O_12_		Antioxidant, antimicrobial antibiolim, anti-inflammatory, anticancer, antidiabetic and immunomodulatory activities	^53,58,65,68^
4	Hexadecanoic acid	11.50	12.3	256.42	C_16_H_32_O_2_		Antioxidant, antimicrobial antibiolim, anti-inflammatory, anticancer, antidiabetic and immunomodulatory activities	^53,58,65,68^
5	Chlorogenic acid	11.92	8.5	354.31	C_16_H_18_O_9_		Antioxidant, antimicrobial antibiolim, anti-inflammatory, anticancer, antidiabetic and immunomodulatory activities	^53,58,65,68^
6	Caftaric acid	13.95	6.5	312.27	C_14_H_14_O_8_		Antioxidant, antimicrobial antibiolim, anti-inflammatory, anticancer, hepatoprotective and immunomodulatory activities	^53,58,65,68^
7	Linoleic acid	18.25	18.7	280.45	C_18_H_32_O_2_		Antioxidant, antimicrobial anti-inflammatory, anticancer, skin protective, wound healing and immunomodulatory activities	^53,58,65^
8	Octadecanoic acid	22.62	9.6	284.48	C_18_H_36_O_2_		Antioxidant, antimicrobial antibiolim, anti-inflammatory, anticancer, antidiabetic and immunomodulatory activities	^53,58,65^
8	Cichoric acid	24.95	7.4	474.37	C_22_H_18_O_12_		Antioxidant, antimicrobial a antiviral, anti-inflammatory,, antidiabetic and immunomodulatory activities	^53,58,65^

## Conclusion

This study highlights the therapeutic potential of *C. intybus* leaf extract against multidrug-resistant Gram-negative pathogens. Clinical isolates were predominantly *K. pneumoniae* (56%), followed by *E. coli* (24%) and *A. baumannii* (20%), exhibiting high resistance to β-lactams, moderate resistance to aminoglycosides and tetracyclines, and retained susceptibility to imipenem and amikacin, underscoring the clinical challenge posed by MDR infections.

Collectively, *C. intybus* extract demonstrated potent and multifaceted bioactivity, including significant antibacterial effects against multidrug-resistant *K. pneumoniae*,* E. coli*, and *A. baumannii*, as well as pronounced, dose-dependent antibiofilm activity at sub-MIC levels, indicating interference with bacterial virulence independent of bactericidal action. These effects were complemented by substantial antioxidant capacity (DPPH: 92.2 ± 1.5%; ABTS: 90.5 ± 2.0%; IC_50_ ≈ 110–115 µg/mL) and selective cytotoxicity toward cancer cell lines (PC3 and HepG2) via apoptosis induction, supported by significantly lower IC_50_ values compared to normal HFB4 cells.

Phytochemical profiling by GC–MS and HPLC revealed the presence of diverse bioactive constituents, including fatty acids, phenolic acids, flavonoids, and terpenoids (e.g., hexadecanoic, octadecanoic, chlorogenic, cichoric, linoleic, and palmitic acids), which likely underpin the observed antimicrobial, antioxidant, and anticancer activities, thereby highlighting the extract’s potential as a promising candidate for therapeutic and pharmaceutical applications.

However, this study is subject to several limitations, including its exclusively in vitro design and the use of a single extraction solvent, which may have restricted the recovery of certain polar phytoconstituents. Furthermore, the relatively small number of isolates employed in functional assays, the absence of stability and shelf-life evaluations, and the lack of investigation into underlying molecular mechanisms constrain the robustness of the findings. The absence of in vivo validation further limits the translational and clinical applicability of the results.

Future studies should focus on mechanistic elucidation, in vivo pharmacological validation, stability profiling, fractionation and isolation of active compounds, and evaluation of synergistic interactions with conventional antibiotics to better support the development of *C. intybus*-based therapeutics against MDR pathogens.

## Data Availability

All data generated or analyzed during this study are included in this published article.
